# The Neural Processes Underpinning Flexible Semantic Retrieval in Visual and Auditory Modalities

**DOI:** 10.1002/hbm.70536

**Published:** 2026-04-22

**Authors:** Ximing Shao, Meichao Zhang, Xiuyi Wang, Andre Gouws, Rebecca L. Jackson, Jonathan Smallwood, Katya Krieger‐Redwood, Elizabeth Jefferies

**Affiliations:** ^1^ Department of Psychology University of York York UK; ^2^ Department of Linguistics and Modern Languages The Chinese University of Hong Kong Hong Kong SAR China; ^3^ CAS Key Laboratory of Behavioral Science, Institute of Psychology Chinese Academy of Sciences Beijing China; ^4^ Department of Psychology Queen's University Kingston Ontario Canada

## Abstract

Contemporary accounts of semantic cognition propose that conceptual knowledge is supported by a heteromodal conceptual store and controlled retrieval processes. However, it remains unclear how the neural basis of semantic control varies across modalities. Recent models of cortical organisation suggest that control networks are distributed along a unimodal‐to‐heteromodal cortical gradient, with the semantic control network (SCN) located in more heteromodal cortex than the domain‐general multiple demand network (MDN). We used fMRI to examine how these networks respond to semantic control demands in visual and auditory tasks. Participants judged the semantic relatedness of spoken and written word pairs. On half of the trials, a task cue specified the semantic feature to guide retrieval; on the remaining trials, no such cue was given. The SCN showed greater activation when task knowledge was available, consistent with a role in the top‐down control of semantic retrieval across modalities. In contrast, the MDN showed greater activation for spoken words, likely reflecting increased demands in speech perception. These findings demonstrate a dissociation between control networks, with SCN involvement modulated by task structure and MDN activity influenced by perceptual difficulty.

## Graphical abstract

Using fMRI, we reveal roles of two distinct frontoparietal networks to semantic control demands. The semantic control network responds to top‐down retrieval goals, while the multiple‐demand network is more active for spoken words, reflecting perceptual‐decisional demands. This dissociation aligns with the positions of these networks along a unimodal‐heteromodal cortical gradient.

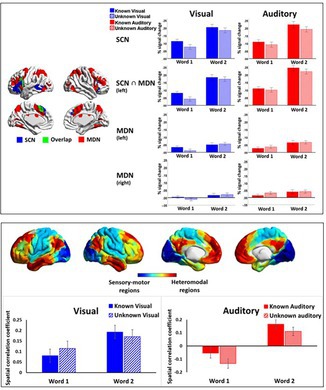

## 1 Introduction

Semantic cognition recruits both unimodal regions for sensory‐motor processing and a heteromodal network that shows common responses across spoken and written words, as well as non‐verbal meaningful materials such as pictures (Jefferies 2013; Lambon Ralph et al. 2017). The Controlled Semantic Cognition account proposes that distinct brain areas have different functions (Jefferies 2013; Lambon Ralph et al. 2017; Patterson et al. 2007; Whitney et al. 2011): bilateral anterior temporal lobe (ATL) acts as a hub supporting long‐term conceptual representation (Patterson et al. 2007), modality‐specific ‘spoke’ cortices encode different types of input information, such as words, pictures, sounds, etc. (Lambon Ralph et al. 2017; Kuhnke et al. 2023; Binder and Desai 2011; Borghesani and Piazza 2017; Kiefer and Pulvermüller 2012), while a left‐lateralised control network interacts with the hub to produce flexible, context‐ and task‐appropriate responses by ensuring that only relevant aspects of semantic representations are used to direct thought and behaviour (Whitney et al. 2011; Noonan et al. 2013; Jackson 2021; Reilly et al. 2024). This semantic control process—which reflects the specific cognitive operations needed to constrain conceptual retrieval to suit current goals and context—is dissociable from the processes underlying response selection and inhibition. For example, judging ‘dog‐cat’ requires little control due to a strong direct association, whereas judging ‘dog–chipmunk’ demands greater control to evaluate a weaker semantic link. Successful semantic retrieval in such contexts requires the dynamic interaction of the representation and control systems to align retrieval with the task goal or context. Both the representation system that supports long‐term concepts and the control system appear to be heteromodal, meaning that these systems involve cortical areas that receive and process converging inputs from multiple sensory and motor systems (Reilly et al. 2024). Patients with semantic dementia have consistent semantic deficits across different input and output systems and different types of tasks, suggesting that they have degradation of ‘central’ crossmodal concepts (Patterson et al. 2007). In contrast, people with semantic aphasia have deregulated semantic cognition affecting both verbal and non‐verbal semantic tasks, such as object use (Corbett et al. 2011); they have difficulty controlling retrieval such that it is appropriate for the task or context, especially when the knowledge that is required is weakly encoded or subject to competition from irrelevant meanings (Jefferies and Lambon Ralph 2006; Jefferies 2013). Neuroimaging studies also show common recruitment of semantic regions across tasks involving spoken and written words and pictures (Binder and Desai 2011; Humphreys et al. 2015; Patterson et al. 2007).

While this research points to the existence of heteromodal semantic regions, it does not preclude the possibility that modality‐related differences are expressed *within* this network. Contemporary views of cortical organisation propose that heteromodal areas are arranged along large‐scale functional gradients, reflecting increasing distance from unimodal sensory and motor cortex (Huntenburg et al. 2018; Margulies et al. 2016). Within this framework, heteromodal regions such as default mode and fronto‐parietal control cortex occupy positions relatively far from primary sensory areas along the cortical surface, a spatial organisation captured by the principal gradient of intrinsic connectivity. Importantly, even within heteromodal semantic systems, graded functional variation has been observed. For example, subregions of the anterior temporal lobes show differences in functional properties depending on their relative proximity to converging sensory–motor inputs (Lambon Ralph et al. 2017). By extension, heteromodal regions that lie closer to particular primary systems along the cortical gradient may exhibit stronger modality‐related effects, reflecting differences in the balance of sensory‐specific versus integrative processing (cf. Wang et al. 2023).

In addition, recent research suggests that multiple, partially dissociable control networks contribute to semantic cognition (Chiou et al. 2023; Davey et al. 2016; Wang et al. 2020; Gao et al. 2021, 2022); these control networks might show differences across modalities. The multiple‐demand network (MDN), centred on the inferior frontal sulcus and intraparietal sulcus, is thought to support goal maintenance and selection of relevant representations across domains (Duncan 2010; Duncan et al. 2020; Fedorenko et al. 2013). In contrast, the semantic control network (SCN), with peaks in the left inferior prefrontal and posterior temporal cortex, responds more selectively to control demands within semantic tasks (Badre and Wagner 2007; Chiou et al. 2018; Davey et al. 2016; Jackson 2021; Jefferies 2013; Lambon Ralph et al. 2017; Noonan et al. 2010; Noonan et al. 2013). These networks have distinct topographies: semantic control processes are highly left‐lateralised (Davey et al. 2016; Gonzalez Alam et al. 2019; Whitney et al. 2011), while MDN is a bilateral network (Duncan 2010; Duncan et al. 2020; Fedorenko et al. 2013). Moreover, SCN is sandwiched between DMN and the multiple demand network (MDN) on the cortical surface, and SCN is closer to the heteromodal end of the principal gradient compared to MDN (Chiou et al. 2023; Davey et al. 2016; Wang et al. 2020), suggesting that MDN might show stronger effects of input modality. Within the left inferior frontal gyrus (LIFG) and dorsomedial prefrontal cortex (DMPFC), there may be functional gradients (Diveica et al. 2023; Jung et al. 2022; Wang et al. 2020), with more anterior and ventral aspects showing stronger connectivity with the default mode network—and more selective recruitment in semantic tasks, while more posterior regions support demanding sensory‐motor as well as cognitive tasks—suggesting they might show greater effects of input modality (Krieger‐Redwood et al. 2015; Jackson 2021).

In this study, we examined how semantic control processes interact with input modality by manipulating task knowledge during spoken and written word judgements. Prior work has shown that top‐down control over semantic retrieval recruits the SCN, particularly LIFG, in the visual domain (Zhang et al. 2021). We extended this approach to the auditory modality to test whether the SCN supports controlled retrieval in a modality‐independent fashion, and to explore how it differs from the domain‐general MDN in response to semantic task demands. Participants judged whether word pairs presented either visually or aurally were semantically related via thematic (e.g., *dog–collar*) or taxonomic (e.g., *dog–chipmunk*) links, or were unrelated (e.g., *walnut–clock*). For the related word pairs, we selected word pairs with moderate semantic relatedness—those sharing a broad superordinate category (e.g., “mammals”) or a thematic link, but which are not among the strongest associations. This allows us to effectively examine the semantic retrieval and control process. For the unrelated condition, we used word pairs for which no plausible association can be made, such as “*walnut–clock*” and “*jacket–coconut*”. On half the trials, a cue indicated the relevant relation type in advance, enabling goal‐directed semantic retrieval; on the remaining trials, no such information was provided. This design allowed us to examine how task knowledge and input modality influence engagement of distinct control networks. The presence of task knowledge enabled us to examine goal‐directed semantic retrieval, while the comparison between word position isolated semantic decision‐making, which involves integrating information across both words and removes single‐word processing. A gradient‐based view of cortical organisation suggests that the SCN, situated in heteromodal cortex, should support controlled semantic retrieval across modalities when goals are specified in advance. In contrast, the MDN, located closer to unimodal sensorimotor cortex, may be more sensitive to modality‐specific processing demands. Recognising isolated spoken words is inherently more ambiguous than reading written text, particularly in the acoustically challenging MRI environment. We therefore expect MDN activity to reflect perceptual effort more than semantic control demands. By orthogonally manipulating task structure and modality, this study tests how domain‐specific and domain‐general control processes are embedded within the brain's large‐scale functional architecture.

## 2 Methods

### 2.1 Design

Two groups of participants performed semantic judgements to visual or spoken words, with and without the opportunity for top‐down control over retrieval. Both groups were presented with two words in succession (Word 1, Word 2); the critical manipulation of top‐down control was achieved by providing a cue word specifying the type of semantic link (Category or Thematic) relevant to the forthcoming judgement on half the trials (Known condition), compared to no such prior knowledge (Unknown condition). The final decision on each trial was whether the two words were semantically linked. This design maps directly onto the analysis factors of Word Position (Word 1 vs. Word 2), Task Knowledge (link Known or Unknown), and Modality (Visual vs. Auditory). The Known vs. Unknown manipulation was designed to manipulate the extent to which top‐down processes can constrain semantic retrieval. This factor of the design can establish whether the SCN activates more when the type of relationship between successive words is known in advance, and therefore semantic retrieval can be directed towards current goals, even though semantic decision making is not more difficult under these circumstances. To examine processing that increases at the semantic decision‐making stage, we analysed the contrast of Word 2 > Word 1. This contrast provides an operational measure of processes engaged at the onset of the second word, including integration and task‐guided evaluation of the semantic relationship between the two concepts, relative to processing associated with single‐word presentation. By examining the main effects of these two contrasts (i.e., Known vs. Unknown; Word 2 vs. Word 1) and their interaction, we are able to distinguish if there are differences in the neural basis of semantic decision‐making and top‐down semantic control when comparing spoken and written words. The data from participants tested on written words were previously published (Zhang et al. 2021) and we collected new data using the same stimuli and paradigm, and employing the same scanner and imaging sequence, to examine similarities and differences between two input modalities (slight adaptations to the methods for auditory inputs are outlined below).

### 2.2 Participants

For the auditory experiment, 32 students were recruited (mean age = 21.9 ± 3.91 years, 5 males). Five participants were excluded from data analysis due to low accuracy on the semantic decision task (Mean ± SD = 57.5% ± 5.69%). Consequently, 27 participants were included in the final analysis. For the visual experiment (Zhang et al. 2021), 32 students were recruited (mean age = 20.6 ± 1.52 years, 5 males). One participant was excluded from data analysis due to low accuracy on the semantic decision task and therefore 31 participants were included in the final analysis. All participants across both groups were right‐handed native English speakers and had normal or corrected‐to‐normal vision. None of them had any history of neurological impairment, diagnosis of learning difficulty, or psychiatric illness.

A separate sample of 176 participants (mean age = 20.57, 114 females) who completed resting‐state fMRI was used to examine the intrinsic connectivity of regions identified in task contrasts. For all samples, ethical approval was obtained from the Research Ethics Committees of the Department of Psychology and York Neuroimaging Centre, University of York. All participants provided written informed consent prior to taking part and received monetary compensation or course credit for their time.

### 2.3 Materials

In both the visual and auditory tasks, participants decided whether the probe and target words were semantically related in a semantic relatedness judgement task. Items were linked by one of two different types of semantic relationships only—taxonomic (i.e., they were in the same semantic category) or thematic (i.e., the items were commonly found or used together). On half of the trials, participants were told in advance which relationship would be probed before the presentation of the word pair. For the other half of the trials, participants decided about semantic relatedness based on the two items presented, with no specific instructions in advance. A 2 (Task Knowledge: Known Goal vs. Unknown Goal) × 2 (Semantic Relation: Taxonomic relation vs. Thematic relation) fully‐factorial within‐subjects design was used to create four conditions, with each experimental condition including 30 related trials. A total of 60 unrelated word pairs were generated without repeating words from the related pairs (i.e., each pair was unique and there was no overlap across conditions). Overall, 120 related and 60 unrelated word pairs were included in this task. We included a greater number of related trials (*n* = 120) to ensure robust modelling and comparison of the BOLD signal across conditions, which is the focus of our hypotheses. The number of unrelated trials (*n* = 60), while smaller, is sufficient to yield a reliable baseline parameter estimate. This asymmetric design allows us to maximise statistical power for the contrasts of interest while keeping the scan session to a prudent length, thereby minimising participant fatigue and maintaining data quality. The trials were then evenly divided into two sets corresponding to the Known Goal (60 related trials, 30 unrelated) and Unknown Goal (60 related trials, 30 unrelated) conditions.

The assignment of words to conditions was confirmed using an independent sample of 30 participants who provided subjective ratings of thematic relatedness (co‐occurrence), taxonomic relatedness (physical similarity), and the difficulty of identifying a connection between the items. Thematically‐related word pairs had higher co‐occurrence compared to taxonomically‐related word pairs, while the taxonomically‐related word pairs had higher physical similarity than the thematically‐related word pairs. Rated difficulty was the same across both the taxonomic and thematic conditions, and across Known Goal and Unknown Goal trials. For unrelated word pairs, another 12 participants rated Co‐occurrence, Physical similarity and Difficulty to confirm the lack of semantic links and equivalence across the Known and Unknown Goal conditions (see Table S2 and Zhang et al. 2021 for detailed statistics). In addition, linguistic properties (i.e., word frequency, length, and imageability) of the probe and target words were matched across conditions (see Zhang et al. 2021 for details). Word2vec was also used to provide a metric of strength of association for each word pair, since Zhang et al. (2021) included this variable as a parametric regressor to investigate the effects of controlled retrieval demands when linking together more weakly related concepts. Word2vec is a measure of semantic distance that is based on the assumption that words with similar meanings occur in similar contexts (Mikolov et al. 2013), and this metric can capture both taxonomic (physical similarity) and thematic (occurrence in similar contexts) relationships (see Zhang et al. 2021 for details).

The auditory task employed the same words as the published visual task to allow their direct comparison (Zhang et al. 2021). All words in the auditory task (including instruction words) were recorded in a female voice using Praat software (www.fon.hum.uva.nl/praat/). Each audio word was supplied as a 16‐bit mono WAV file at a sampling rate of 44,100 Hz, and the intensity of each word was processed uniformly to 70 dB. The volume of the spoken words output from the headphones was set to a safe level.

Both the visual and auditory scanning sessions also included a non‐semantic baseline task. The non‐semantic baseline task was included to account for low‐level sensory processing and motor output, allowing us to isolate the neural correlates specific to semantic cognition beyond these fundamental processes. In the visual session, participants were presented with a pair of meaningless letter strings in succession and were asked to decide if they contained the same number of letters (full details in Zhang et al. 2021). In the auditory session, one spoken number was presented as the probe, followed by two spoken numbers in succession. Participants were asked to decide whether the probe number was equal to the sum of the other two numbers. In both the visual and auditory sessions, there were 30 matching trials and 15 mismatching trials in the baseline task.

### 2.4 Procedure

**FIGURE 1:**
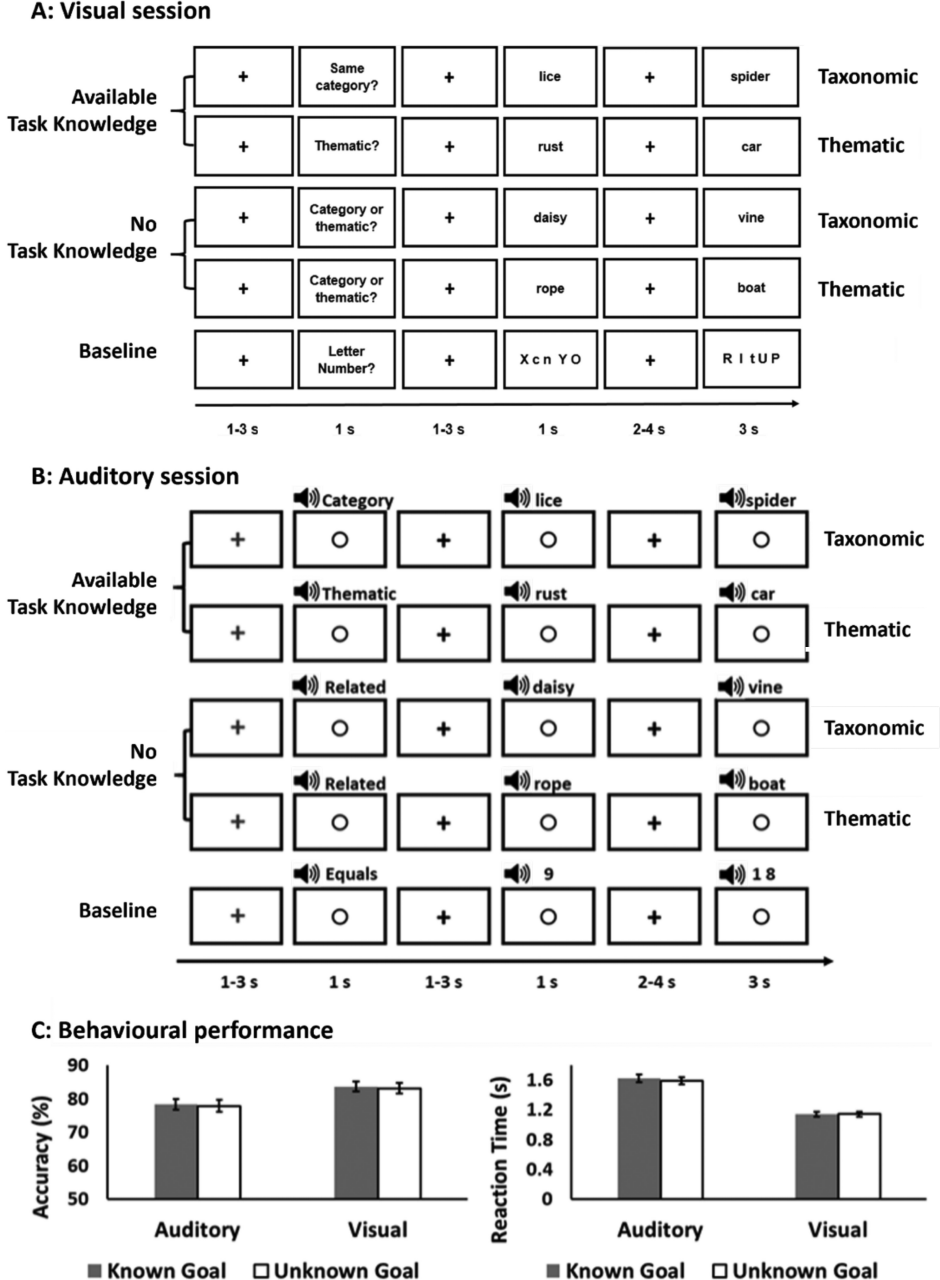
Task design and behavioural data. (A) Illustration of visual task, adapted from Zhang et al. (2021); (B) Illustration of auditory task. (C) Behavioural results (mean accuracy and reaction time) for auditory and visual modalities. Error bar represents mean standard error.

In both the auditory and visual sessions, participants were asked to decide if the words in each pair were semantically related (i.e., either from the same category or thematically related) or unrelated. We manipulated the opportunity for top‐down controlled semantic retrieval by changing the instructions before the word pair was presented. On half of the trials, participants were presented with a specific task instruction (‘Category?’ or ‘Thematic?’) before the word pair, so that they knew in advance which type of semantic relationship would be relevant on the trial (Known Goal). On the other half of the trials, participants heard a non‐specific instruction (‘Related?’) ahead of the word pair, and they made the decision only from the stimuli alone. The manipulation of task knowledge (i.e., Known Goal vs. Unknown Goal) allowed us to examine whether there is a different response in the brain when participants knew the type of semantic relation between the words in advance or not. In the non‐semantic baseline condition, participants in the visual session saw the instruction ‘Letter Number’ followed by two meaningless letter strings, while participants in the auditory session heard ‘Equals?’ as the task instruction, followed by a spoken number (e.g., ‘9’) as the probe, and two spoken numbers presented in quick succession (e.g., ‘1, 8’) as the target to sum (see Figure 1A,B for details).

Figure 1A,B indicate the procedure for each condition; this was consistent across written and spoken sessions (Zhang et al. 2021). The stimulus onset timings were held constant across visual and auditory sessions; all audio words (including task instructions, probes and targets) were shorter than 1 s, and were played synchronously with a visual cue (a circle ‘O’ in the centre of the screen). Each trial started with a red fixation cross presented for a jittered interval of 1–3 s in the centre of the screen. Then the task instruction was presented, followed by a jittered inter‐stimulus fixation for 1–3 s. Then the probe item was presented. After a longer jittered fixation interval lasting 2–4 s, the target item was presented during a 3 s decision window. Participants pressed one of two buttons on a response box to indicate whether the words were semantically related (or to respond to the non‐semantic baseline) as fast and accurately as possible. They used their right index and middle fingers to indicate YES and NO responses. In the auditory condition, if they could not hear the word pair clearly, they pressed a button with their ring finger to indicate this. The ratio of YES to NO responses was held constant across experimental conditions. In both sessions, stimuli were presented in five runs each containing 45 trials: 6 related and 3 unrelated trials in each of the four experimental conditions, and 9 non‐semantic baseline trials. Each run lasted 9 min, and trials were presented in a random order. The runs were separated by a short break and started with a 9‐s alerting slide (i.e., Experiment starts soon).

### 2.5 Neuroimaging Data Acquisition

Structural and functional data were acquired using a 3 T GE HDx Excite Magnetic Resonance Imaging (MRI) scanner utilising an eight‐channel phased array head coil at the York Neuroimaging Centre, University of York. Structural MRI acquisition was based on a T_1_‐weighted 3D fast spoiled gradient echo sequence (repetition time (TR) = 7.8 s, echo time (TE) = minimum full, flip angle = 20°, matrix size = 256 × 256, 176 slices, voxel size = 1 × 1 × 1 mm). Task‐based activity was recorded in the same way in the visual and auditory sessions. We used single‐shot 2D gradient‐echo‐planar imaging sequence with TR = 3 s, TE = 35 ms, flip angle = 90°, matrix size = 64 × 64, 45 slices, and voxel size = 3 × 3 × 3 mm. Data for each modality was acquired in a single session. The task was presented across five functional runs, each containing 185 volumes.

In an independent sample, a 9‐min resting‐state fMRI scan was recorded using single‐shot 2D gradient‐echo‐planar imaging (TR = 3 s, TE = 35 ms, flip angle = 90°, matrix size = 64 × 64, 60 slices, voxel size = 3 × 3 × 3 mm, 180 volumes). The participants were instructed to focus on a fixation cross with their eyes open and to keep as still as possible, without thinking about anything in particular. These data have been used in previous studies (e.g., Evans et al. 2020; Gonzalez Alam et al. 2019; Shao et al. 2022; Vatansever et al. 2017; Wang et al. 2018).

### 2.6 Pre‐Processing of Task‐Based fMRI Data

All functional and structural data were pre‐processed using a standard pipeline and analysed via the FMRIB Software Library (FSL version 5.0, www.fmrib.ox.ac.uk/fsl). Individual T_1_‐weighted structural brain images were extracted using FSL's Brain Extraction Tool (BET). Structural images were linearly registered to the MNI152 template using FMRIB's Linear Image Registration Tool (FLIRT). The first three volumes (i.e., the presentation of the 9‐s task reminder ‘Experiment starts soon’) of each functional scan were removed to minimise the effects of magnetic saturation. The functional data were analysed by using FSL's FMRI Expert Analysis Tool (FEAT). We applied motion correction using MCFLIRT (Jenkinson et al. 2002), slice‐timing correction using Fourier space time‐series phase‐shifting (interleaved), spatial smoothing using a Gaussian kernel of FWHM 6 mm, and high‐pass temporal filtering (sigma = 100 s) to remove temporal signal drift. In addition, motion scrubbing (using the fsl_motion_outliers tool) was applied to exclude volumes that exceeded a framewise displacement threshold of 0.9 (Siegel et al. 2014; Power et al. 2012).

### 2.7 Univariate Analysis of Task‐Based fMRI Data

The univariate analysis examined the controlled retrieval of semantic information at different stages of the task. The general linear model (GLM) was constructed in an identical fashion for the visual and auditory modalities, following Zhang et al. (2021), to allow a direct comparison of these sessions. We differentiated the response to the first word, when participants accessed semantic representations and prepared for either a specific type of semantic relationship (Specific goal: ‘Category?’ or ‘Thematic?’) or a judgement of either type (Non‐specific goal: ‘Related?’), from the second word when participants made a decision about whether the words were linked according to the specific instructions or in the absence of a specific instruction about the nature of the semantic relationship.

Consequently, the model included three factors: (1) Word Position (First word vs. Second word), (2) Task Knowledge (Known Goal vs. Unknown Goal), and (3) Semantic Relation (Taxonomic relation vs. Thematic relation). In addition, we included word2vec (Mikolov et al. 2013) as a parametric regressor in the model to characterise the semantic distance between the two words in each trial, in line with the previous study employing written words (Zhang et al. 2021). There were no effects of word2vec at the whole‐brain level in the current dataset employing spoken words.

The pre‐processed time‐series data were modelled using a general linear model, using FMRIB's Improved Linear Model (FILM) correcting for local autocorrelation (Woolrich et al. 2001). 10 Explanatory Variables (EV) of interest and 5 of no interest were modelled using a double‐Gaussian hemodynamic response gamma function (probe and target were modelled separately, therefore there were two EVs for each condition). The 10 EVs of interest were: (1) Probe and (2) Target for Known Goal Taxonomic Relations, (3) Probe and (4) Target for Unknown Goal Taxonomic Relations, (5) Probe and (6) Target for Known Goal Thematic Relations, (7) Probe and (8) Target for Unknown Goal Thematic Relations, (9) Probe and (10) Target for baseline condition (for matching trials, since the semantic data only included related trials). Our EVs of no interest were: (11) Probe and (12) Target for unrelated word pairs, (13) Other inputs‐of‐no‐interest (including the audio instruction word, the period after the response on each trial, and mismatching baseline trials), (14) Fixation (post‐task instruction and within‐trial fixations were modelled to account for task preparation and automatic retrieval process, respectively, while between trial fixations remained as the implicit baseline), and (15) Incorrect Responses (including all the incorrect trials across conditions, and the trials where participants indicated the auditory stimulus was unclear). We also included an EV to model word2vec as a parametric regressor, therefore we had 16 EVs in total. EVs for the first item in each pair commenced at the onset of the audio word or number, with EV duration set as the presentation time (1 s). EVs for the second item in each pair commenced at the onset of the written or spoken word or number, and ended with the participants' response (i.e., a variable epoch approach was used to remove effects of time on task). The remainder of the second item presentation time was modelled in the inputs‐of‐no‐interest EV (i.e., in the visual task, word 2 was on screen for 3 s, and if a participant responded after 2 s, the post‐response period lasting 1 s was removed and placed in the inputs‐of‐no‐interest EV). The parametric word2vec EV had the same onset time and duration as the EVs corresponding to the second word in the semantic trials, but included the demeaned word2vec value as a weight. The fixation period between the trials provided the implicit baseline. The potential temporal overlap of the hemodynamic responses evoked by Word 1 and Word 2 is addressed through our experimental design and analytical model. By employing a jittered inter‐stimulus interval, the predicted BOLD signals for the two events are partially orthogonalized, which enhances the efficiency with which the general linear model (GLM) can estimate their separate contributions (Dale 1999; Friston et al. 1999). Consequently, the combined approach of a jittered, event‐related design and the inclusion of distinct regressors for each word position within a single GLM constitutes a standard and validated methodological framework for isolating neural activity associated with sequentially proximate cognitive events (Burock et al. 1998; Poldrack et al. 2011). The correlations between condition regressors were all sufficiently independent (most |*r*| values below 0.1 and almost all |*r*| values below 0.4). Group‐averaged correlation matrices for auditory and visual modalities among all EVs are provided in Tables S3–S4 and Figures 3 and 4.

The five sequential runs were combined using fixed‐effects analyses for each participant. In the higher‐level analysis at the group level, the combined contrasts were analysed using FMRIB's Local Analysis of Mixed Effects (FLAME1), with automatic outlier de‐weighting (Woolrich 2008). A 50% probabilistic grey‐matter mask was applied. Clusters were thresholded using Gaussian random‐field theory, with a cluster‐forming threshold of *z* = 3.1 and a familywise‐error‐corrected significance level of *p* = 0.05.

In addition to contrasts examining the main effects of Word Position (Word 1 vs. Word 2), and Task Knowledge (Known Goal vs. Unknown Goal) for both modalities, we included two‐way interaction terms of Modality by Task Knowledge, as well as Modality by Word Position. These two‐way interactions were tested at the group level using a mixed‐effects model (FLAME 1) in FSL's FMRI Expert Analysis Tool (FEAT), with Modality specified as a between‐subjects factor, and Task Knowledge/Word position as within‐subjects factors. We also included contrasts of all task conditions between visual and auditory modalities (i.e., Known Goal Thematic; Known Goal Taxonomic; Unknown Goal Thematic; Unknown Goal Taxonomic comparing spoken and written words).

### 2.8 Selection of ROIs: Semantic Control Sites and MDN Network

Additional ROI analyses characterised the effects of word position and task knowledge in a priori semantic control and domain‐general executive networks. Using FSL's Featquery (Woolrich et al. 2001), we extracted the percent signal change for each ROI by converting the parameter estimates and averaging across all voxels within our selected networks or semantic control sites. We examined the SCN using a binarised mask from a recent meta‐analysis of semantic control (Jackson 2021). Domain‐general executive regions of the MDN were identified as regions showing difficulty effects across multiple tasks (map taken from Fedorenko et al. 2013). These functionally‐defined control networks are partially overlapping, especially in the left hemisphere, although they also have distinct elements—with semantic control extending to more anterior and ventral parts of left inferior frontal gyrus, and left posterior temporal cortex, while MDN is focused on bilateral inferior frontal and intraparietal sulcus. We created four functional network masks from the combination of these network maps: (i) MDN regions also implicated in semantic control (i.e., SCN ∩ MDN), (ii) SCN‐only regions not implicated in domain‐general control, (iii) MDN‐only regions not implicated in semantic control in the left hemisphere, (iv) MDN‐only regions in the right hemisphere. These maps are available on Neurovault (https://neurovault.org/collections/14750/). In a supplementary analysis, we also present results for each of the five strongest clusters in the SCN meta‐analysis (Jackson 2021).

### 2.9 Spatial Correlation Between Individual‐Level Maps From the Univariate Analysis and Whole‐Brain Cortical Gradients

We took non‐thresholded maps of each task condition (contrasted against the implicit baseline) at the individual level, and computed the spatial correlation of these maps with the first three cortical gradients derived from intrinsic connectivity (group maps taken from Margulies et al. 2016). This method provides a metric denoting how similar each participant's pattern of activation and deactivation is to key functional dimensions of cortical organisation. Higher positive spatial correlations with Gradient 1 suggest functional responses that are more heteromodal, while negative correlations mean greater similarity with unimodal regions. Repeated‐measures ANOVAs were used to examine whether there were effects of task knowledge, word position, or modality on the location of task activation in gradient space.

## 3 Results

### 3.1 Behavioural Results

Trials with incorrect responses were excluded from the RT analysis (16.3% in the visual modality; 19.1% reported as not heard clearly and 14.5% incorrect responses on clear trials in the auditory modality). Accuracy was calculated as the percentage of correct responses relative to the total number of trials in which the stimulus was heard clearly. Repeated‐measures ANOVAs were performed on both accuracy and RT, examining the effects of Task Knowledge (Known Goal vs. Unknown Goal) and modality (visual vs. auditory).^1^ Figure 1C shows that the auditory task had lower accuracy (left panel, *F*(1, 56) = 6.96, *p* = 0.011, *η*_*p*_^2^ = 0.11) and longer reaction times (right panel, *F*(1, 56) = 71.47, *p* < 0.001, *η*_*p*_^2^ = 0.56). There were no differences in performance for Known Goal versus Unknown Goal trials (Accuracy: *F*(1, 56) = 0.24, *p* = 0.63, *η*_*p*_^2^ = 0.004; RT: *F*(1, 56) = 2.39, *p* = 0.13, *η*_*p*_^2^ = 0.04) and no interaction (Accuracy: *F*(1, 56) = 0.01, *p* = 0.94, *η*_*p*_^2^ = 0.00; RT: *F*(1, 56) = 2.30, *p* = 0.14, *η*_*p*_^2^ = 0.04). These behavioural results suggest that while the effect of task knowledge (i.e., top‐down control) was equivalent for the two modalities, the auditory task was harder overall because the individual items were more difficult to recognise. Therefore, two types of controlled processing may occur within this task: task knowledge is thought to modulate participants' capacity to control semantic retrieval in a top‐down fashion to suit the task demands (Zhang et al. 2021), while individual spoken words are harder to recognise than written words from the input signal.

### 3.2 Univariate Analysis

#### 3.2.1 Activation for Auditory and Visual Semantic Decisions

**FIGURE 2:**
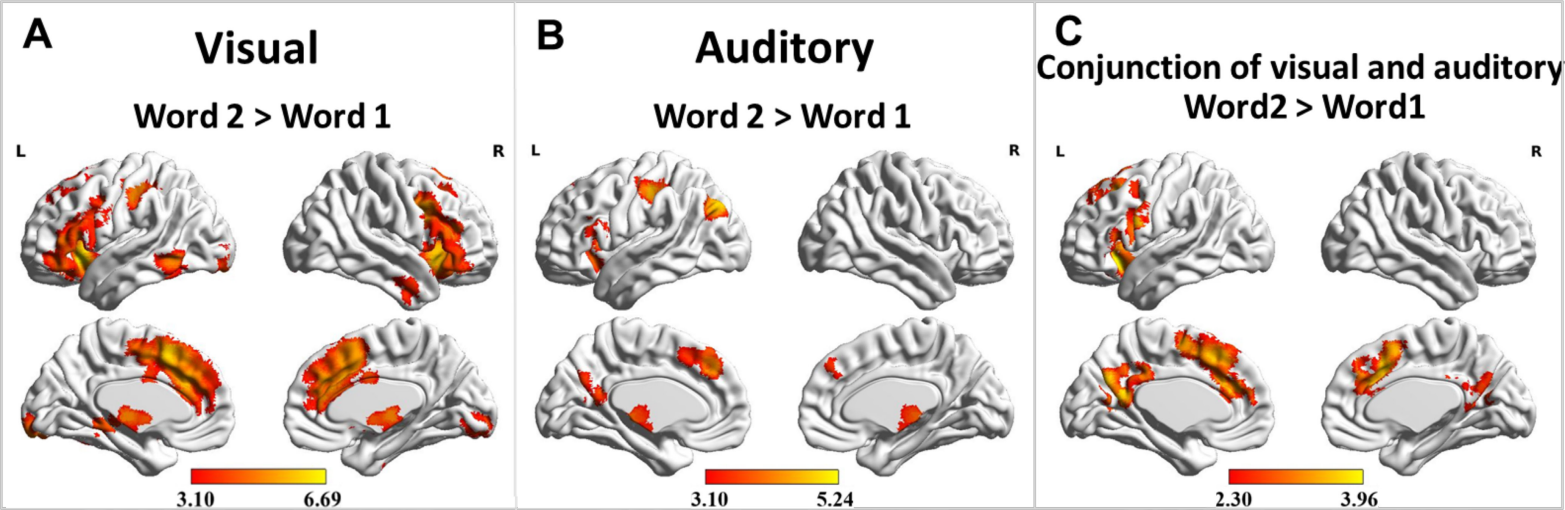
Effect of word position (i.e., the effect of decision‐making) in the visual (A) and auditory modality (B), and regions activated during semantic decisions for both modalities (C). Maps in panels A and B were cluster‐corrected with a voxel inclusion threshold of *z* > 3.1 and family‐wise error rate using random field theory set at *p* < 0.05. The conjunction map in panel C is identified using FSL's ‘easythresh_conj’ tool and thresholded at *z* = 2.3.

First, we examined the brain regions engaged in the semantic decision‐making phase in a whole‐brain analysis, in the visual and auditory modalities respectively. To isolate the demands specific to the semantic decision‐making phase, we examined the contrast of Word 2 > Word 1. This contrast identifies additional cognitive processes required by the Word 2 phase—that is the top‐down evaluation of the semantic relationship between the two words and response selection—beyond basic word recognition and initial semantic retrieval engaged by both words. We conducted a conjunction analysis to investigate the regions recruited in common across written and spoken words (Figure 2). A more liberal threshold (*z* = 2.3) was used to identify the full extent of shared neural resources across modalities, even if the activation in one modality alone does not survive the stricter (*z* = 3.1) correction. Semantic decisions to written words (i.e., significant clusters for the contrast of Word 2 > Word 1 for visually presented words) included bilateral inferior frontal gyrus (IFG), prefrontal cortex, occipital pole, left inferior temporal gyrus (ITG) and precentral cortex. Semantic decisions to spoken words (i.e., significant clusters for the contrast of Word 2 > Word 1 in the auditory domain) included left IFG, lateral occipital cortex and precentral cortex. A conjunction of these maps for written and spoken words showed common activation across modalities in left IFG and dorsolateral prefrontal cortex, pre‐supplementary motor area and posterior cingulate cortex.

The global contrast between visual and auditory tasks, regardless of word order, is shown in Figure S1. Spoken words elicited more activation in and around bilateral auditory cortex and supplementary motor area. Written words elicited more activation in posterior ITG and fusiform cortex close to the visual word form area, posterior intraparietal sulcus, frontal eye fields, and frontal pole.

#### 3.2.2 The Interaction Between Word Position and Modality

Next, we considered whole‐brain differences between auditory and visual modalities in the effect of (i) word position (contrasts of words 1 and 2) and (ii) task knowledge. These analyses can establish if there are differences in the neural basis of semantic decision‐making and top‐down semantic control when comparing spoken and written words.

**FIGURE 3:**
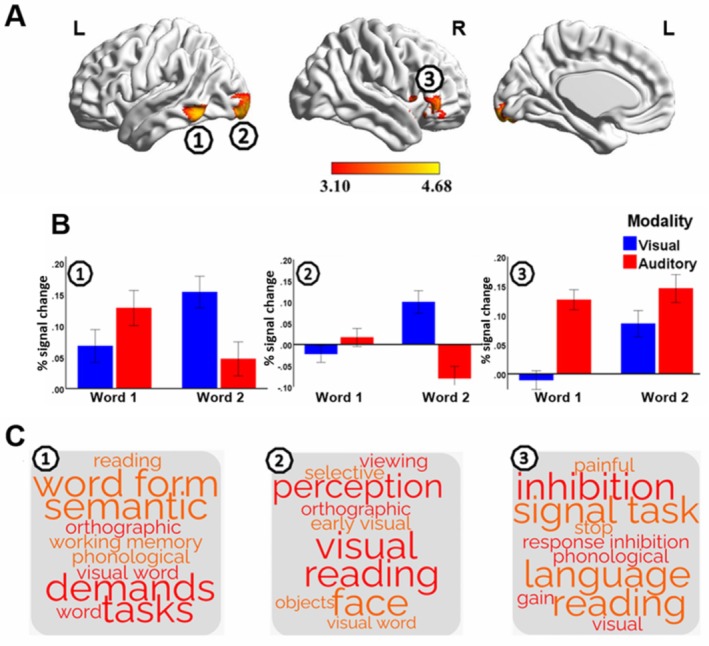
Differences between modalities in the effect of word position. (A) Three clusters were identified in this whole‐brain analysis of the interaction between modality and word position; maps are cluster‐corrected at a threshold of *z* > 3.1 (FWE corrected *p* < 0.05). (B) The signal change within each cluster. (C) Decoding was performed on the unthresholded intrinsic connectivity maps seeded from the three clusters and presented in a word cloud for each cluster.

The interaction of modality with word position revealed three clusters, in left ventral posterior temporal cortex, left occipital pole, and right inferior frontal gyrus (Figure 3A). To examine the role of each cluster, we determined their resting‐state functional connectivity and functionally decoded these unthresholded maps using Neurosynth (Yarkoni et al. 2011). The top 10 relevant terms corresponding to the three clusters were chosen and rendered as word clouds (shown in Figure 3C). Ventral posterior temporal cortex (cluster 1 in Figure 3A) was activated by both written and spoken words, but the effect of word position was stronger for the visual task. Text and speech showed opposite patterns, with written words showing more activation during decision‐making (word 2 > word 1), and spoken words showing more activation for the first word in the pair (when word recognition was more challenging). The connectivity pattern of this cluster was related to terms such as ‘working memory’, ‘word’, and ‘semantic’.

Occipital pole (cluster 2 in Figure 3) showed relatively little response to the first word of the pair in either modality but activated to visual decision‐making, while deactivating in response to auditory decision‐making. The connectivity of this cluster was associated with visual processing and reading.

Finally, the cluster in the right IFG (cluster 3 in Figure 3) showed a significantly stronger response to the auditory task on the first word, when the item was hardest to recognise. This auditory dominance persisted for the second word, although the response difference between modalities was attenuated due to an increased response to the visual stimulus. The connectivity pattern of this cluster was associated with both executive and language processes. These results might therefore reflect stronger engagement of control mechanisms in both auditory speech recognition and in semantic decision‐making.

#### 3.2.3 The Interaction Between Task Knowledge and Modality

**FIGURE 4:**
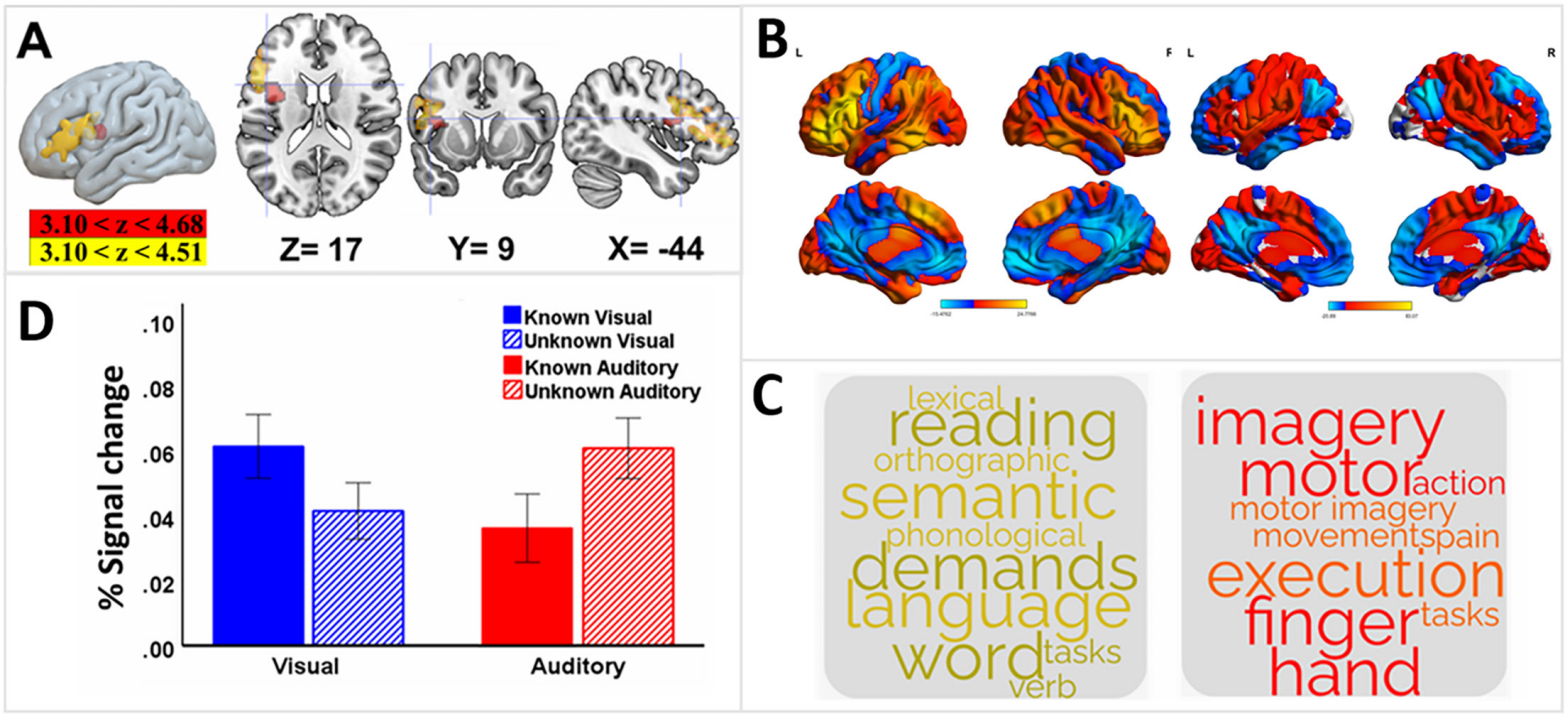
Differences between modalities in the effect of task knowledge. (A) One cluster bordering left posterior insula and IFG (cluster in red colour) was identified in a whole‐brain interaction between modality and task knowledge. This interaction effect was adjacent to the effect of task knowledge in the visual modality (Known Goal > Unknown Goal; cluster in yellow colour). All maps are cluster‐corrected at a threshold of *z* > 3.1 (FWE corrected *p* < 0.05). (B) Unthresholded maps reflecting the intrinsic connectivity of task knowledge in the visual modality (left) and the interaction between modality and task knowledge (right). (C) Decoding results on the unthresholded maps reflecting the effect of task knowledge in the visual modality (B, left) and the interaction between modality and task knowledge (B, right). (D) Signal change within the interaction cluster (red cluster in panel A) in written and spoken tasks respectively. Error bars represent mean standard error.

Next, we examined the interaction of modality with task knowledge, revealing differences across visual and auditory inputs in the effect of prior knowledge about the nature of the semantic relationship to be retrieved. Effects of task knowledge for visual inputs were identified in left IFG by Zhang et al. (2021); yellow in Figure 4A. However, we did not observe similar effects for the auditory modality, and there were no main effects of task knowledge in the whole‐brain analysis. There was a modality by task knowledge interaction in a cluster bordering the left IFG and insula (the red cluster in Figure 4A; signal change within this cluster is shown in Figure 4D), which reflected a stronger effect of task knowledge in the visual domain. We used Neurosynth (Yarkoni et al. 2011) to decode the unthresholded connectivity maps of the visual task knowledge effect (yellow in Figure 4A) and the modality by task knowledge interaction (red in Figure 4A). This approach provides a data‐driven, meta‐analytic interpretation by identifying the terms most consistently associated with each map's spatial pattern, thereby clarifying the cognitive functions linked to these connectivity profiles. The top 10 obtained terms are displayed in a weighted visual format, where the font size of each term corresponds to its degree of association with the decoded connectivity map. The connectivity maps are available on Neurovault (https://neurovault.org/collections/14750/). The top 10 terms obtained from cognitive decoding of the connectivity patterns of these clusters revealed terms such as ‘word’ and ‘semantic’ for Zhang et al.'s task knowledge main effect for written words (yellow in Figure 4A) and ‘motor’ and ‘movement’ for the interaction of modality and task knowledge (red in Figure 4A). In the interaction cluster, written words showed a stronger response when task knowledge was available, while spoken words showed a stronger response without task knowledge. This might reflect differences in the source of difficulty across these two modalities: for the auditory modality there is more uncertainty about the identity of individual words, especially in the absence of task knowledge, while for written words, the most difficult aspect of the task involves establishing a semantic link between the words. Both of these aspects of controlled semantic cognition could involve this region implicated in motor processing. The whole‐brain interactions between modality and word position/task knowledge suggest there are different kinds of task demands operating in our paradigm.

### 3.3 Situating the Individual‐Level Maps From Univariate Analysis in the Whole‐Brain Principal Gradient

**FIGURE 5:**
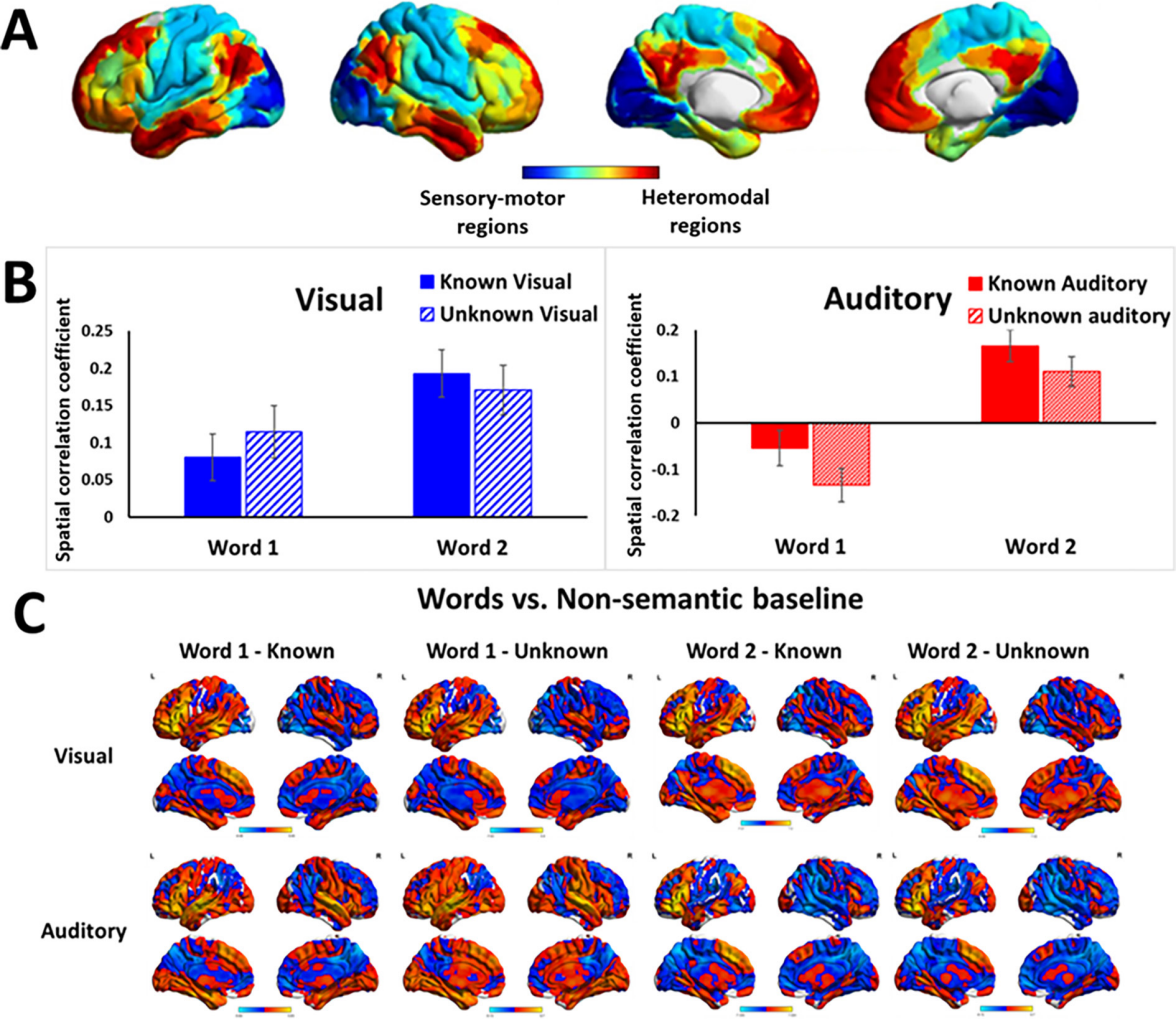
(A) Spatial correlations between the unthresholded individual‐level contrast maps against non‐semantic baseline and the principal gradient derived from resting‐state functional connectivity (Margulies et al. 2016). (B) The spatial correlations for the known and unknown conditions contrasted to non‐semantic baseline at each word position. (C) The unthresholded maps for the known and unknown conditions contrasted to non‐semantic baseline.

To investigate the extent to which *whole‐brain* responses to written and spoken words reflect a different balance of processing between abstract/heteromodal and sensory‐motor regions, and how these differences depend on the availability of task knowledge, we computed spatial correlations between the unthresholded individual‐level contrast maps of each condition (compared with the non‐semantic baseline) and the whole‐brain principal gradient (from Margulies et al. 2016), which captures the distinction in connectivity between unimodal and heteromodal regions. The principal gradient is correlated with distance from primary sensory‐motor landmarks and describes the sequence of resting‐state networks seen along the cortical surface, from sensory‐motor, through attention and frontoparietal networks to the default mode network in multiple areas of cortex (Figure 5A). Positive correlations reveal functional responses that are relatively heteromodal, while negative correlations suggest a stronger response towards unimodal cortex.

An ANOVA of correlations with the principal gradient revealed significant main effects of Modality (*F*(1, 56) = 12.02, *p* = 0.001, *η*_*p*_^2^ = 0.18), Word Position (*F*(1, 56) = 28.31, *p* < 0.001, *η*_*p*_^2^ = 0.34) and Task Knowledge (*F*(1, 56) = 8.87, *p* = 0.004, *η*_*p*_^2^ = 0.14), reflecting a more heteromodal response for written than spoken words, for Word 2 versus Word 1, and for Known compared with Unknown Goal trials (see Figure 5B). The two‐way interactions between Task Knowledge and Modality (*F*(1, 56) = 14.35, *p* < 0.001, *η*_*p*_^2^ = 0.20) and between Word Position and Modality (*F*(1, 56) = 4.80, *p* = 0.033, *η*_*p*_^2^ = 0.08) were also significant. The three‐way interaction was not significant. Follow‐up ANOVAs showed effects of Word Position for both spoken (*F*(1, 26) = 20.64, *p* < 0.001, *η*_*p*_^2^ = 0.44) and written words (*F*(1, 30) = 6.94, *p* = 0.013, *η*_*p*_^2^ = 0.19): the decision‐making phase at Word 2 fell more towards heteromodal regions compared with Word 1 for both modalities. Follow‐up ANOVAs also showed effects of Task Knowledge for auditory (*F*(1, 56) = 21.42, *p* < 0.001, *η*_*p*_^2^ = 0.28) but not visual trials (*F*(1, 56) = 0.352, *p* = 0.555, *η*_*p*_^2^ = 0.006). In the auditory modality, there was a main effect of Task Knowledge (*F*(1, 26) = 15.97, *p* < 0.001, *η*_*p*_^2^ = 0.38) with Unknown Goal trials eliciting a stronger response for spoken words towards sensorimotor regions. In this way, analysis of the activation pattern along the principal gradient found that written words elicited a pattern of activation that was more heteromodal, especially during decision‐making and when task goals were known: these circumstances strengthened the contribution of heteromodal processes to the task state as a whole. In contrast, the balance of processing for spoken words was tilted towards unimodal processes potentially reflecting the relative difficulty of input processing in the auditory task and/or the engagement of control mechanisms focused on sensory‐motor processes.

### 3.4 ROI Analysis to Characterise Effects of Task Knowledge and Word Position in SCN and MDN

The analysis above demonstrates that the balance of unimodal to heteromodal processes is influenced by task knowledge in a different way across modalities. The next analysis considers activation within control systems: although both SCN and MDN are typically thought to be ‘heteromodal’, recent research has shown that these networks occupy different positions on the principal gradient: SCN is closer to the heteromodal end of this gradient than MDN (Chiou et al. 2023; Wang et al. 2020), consistent with the abstract and heteromodal nature of semantic processing, and in line with our observation that SCN responds to task knowledge across modalities, while MDN responds during difficult auditory perception.

Given our results suggest different control demands operate in our task—with input processing being more challenging for spoken words (on word 1, in the absence of task knowledge), while top‐down control can be applied more readily when task knowledge is available (on word 2)—we tested for these different effects of control within four networks of interest: (i) the semantic control network, which is largely left lateralised (SCN, overlap with MDN was excluded), (ii) the overlap of SCN and MDN, which is again largely left lateralised (SCN∩MDN), (iii) the regions of the MDN which do not overlap the SCN in the left hemisphere (MDN left), and (iv) in the right hemisphere (MDN right).

All sites showed main effects of word position with higher activation for the second word (SCN: *F*(1, 56) = 90.94, *p* < 0.001, *η*_*p*_^2^ = 1.00; SCN∩MDN: *F*(1, 56) = 143.62, *p* < 0.001, *η*_*p*_^2^ = 0.72; MDN left: *F*(1, 56) = 17.44, *p* < 0.001, *η*_*p*_^2^ = 0.24; MDN right: *F*(1, 56) = 6.70, *p* = 0.012, *η*_*p*_^2^ = 0.11) and no interaction between modality and *word position*. However, the networks showed different effects of task knowledge. SCN and SCN∩MDN showed main effects of task knowledge (more activation with a Known Goal (SCN: *F*(1, 56) = 13.30, *p* < 0.001, *η*_*p*_^2^ = 0.95; SCN∩MDN: *F*(1, 56) = 10.17, *p* = 0.002, *η*_*p*_^2^ = 0.15)) and no interaction between modality and task knowledge. MDN (SCN∩MDN and MDN in right hemisphere) showed modality effects with higher activation for the harder auditory task (SCN∩MDN: *F*(1, 56) = 8.83, *p* = 0.004, *η*_*p*_^2^ = 0.14; MDN right: *F*(1, 56) = 4.45, *p* = 0.039, *η*_*p*_^2^ = 0.07; this pattern was not found for left MDN). These results suggest that MDN is more important for controlled sensory–motor processing, including the identification of word meaning from spoken inputs in a noisy environment, while SCN is more relevant to top‐down semantic control from goals or expectations.

**FIGURE 6:**
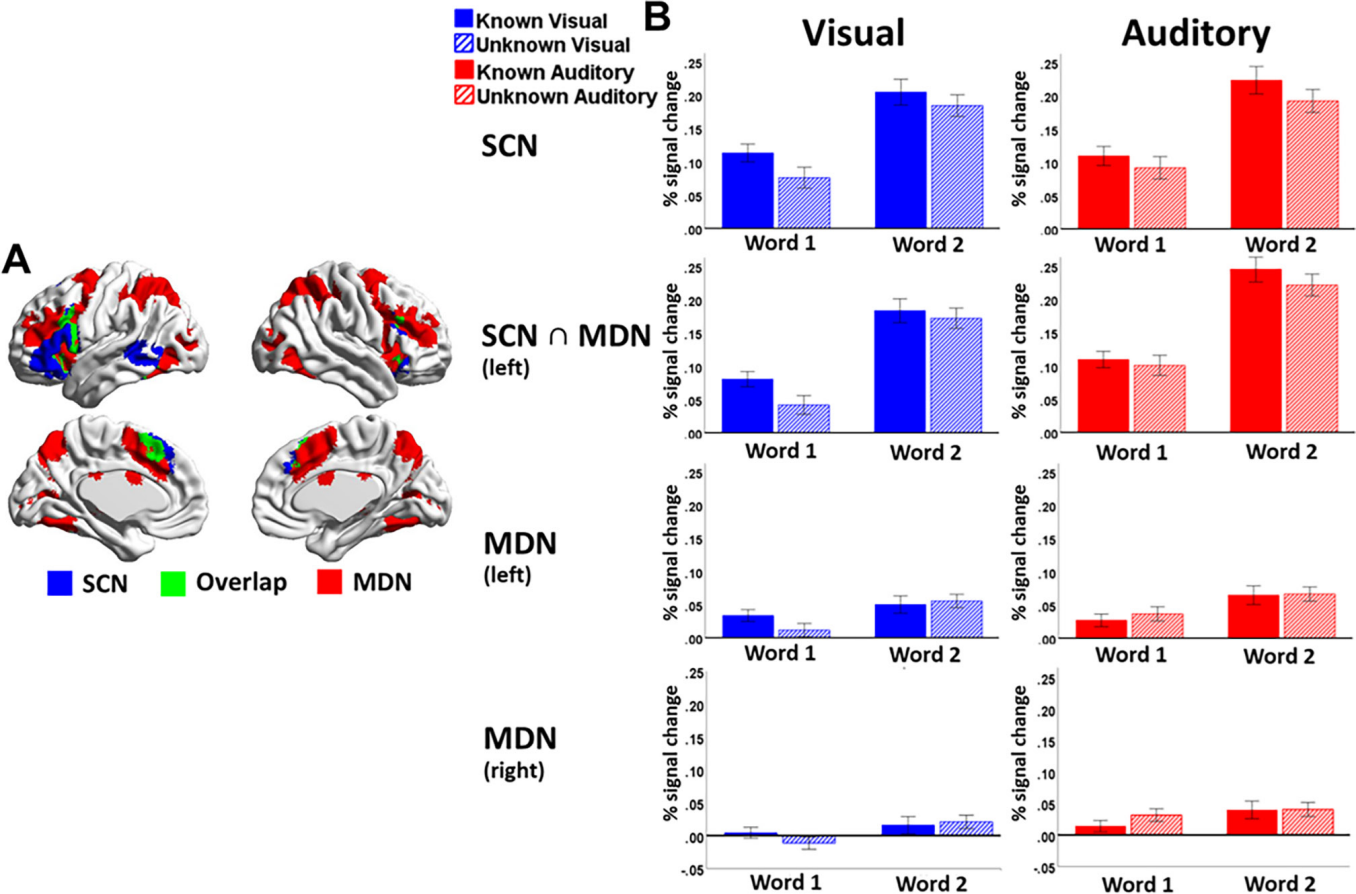
ROI analysis examining the engagement of SCN and MDN. (A) Four ROIs: SCN network (excluded the overlap with MDN, shown in blue), overlap of SCN and MDN (in left hemisphere, shown in green), MDN network (excluding overlap with SCN, shown in red) in left and right hemispheres. (B) ROI analysis for the known and unknown conditions at each word position.

Figure S2 and Table S1 show these effects for the strongest five clusters of the SCN separately (including voxels that also fall within MDN). All sites showed effects of word position with higher activation for Word 2. Most sites (except left pMTG) showed effects of task knowledge with higher activation for Known Goal trials. Effects of modality were inconsistent, with key left‐hemisphere nodes in left IFG and pMTG showing a heteromodal response that was equivalent across visual and auditory inputs, and anterior and posterior right IFG and dorsomedial prefrontal cortex (dmPFC) also implicated in domain general control (see Figure 6A) showing a stronger response to spoken words.

## 4 Discussion

The current study investigated the effect of modality on semantic control by manipulating task knowledge in spoken versus written semantic judgements. The decision‐making phase of the task, across both visual and auditory inputs, elicited activation in heteromodal brain areas implicated in cognitive control and semantic processing. Nevertheless, we identified different kinds of task demands—linked to top‐down effects of task knowledge and the bottom‐up difficulty of identifying spoken words—which dissociated across control networks relevant to semantic cognition (SCN and MDN).

Across modalities, we observed a dissociation between two forms of task demand that mapped onto distinct control networks. SCN showed stronger activation when task goals could be applied in a top‐down manner, irrespective of modality, whereas MDN was more strongly engaged by spoken inputs, which were behaviourally more difficult to process. This pattern aligns with growing evidence that SCN supports controlled semantic retrieval, while MDN is more sensitive to effortful engagement with externally demanding inputs (Chiou et al. 2023; Gao et al. 2021, 2022; Hodgson et al. 2024). Importantly, these functional differences were also captured by the principal gradient of cortical connectivity. Written words elicited greater activation toward the heteromodal end of the gradient, particularly during semantic decision‐making and when task goals were known in advance, whereas spoken words were associated with activation closer to the unimodal end. This suggests that semantic decisions based on written input rely more heavily on heteromodal control processes, while spoken input places greater demands on sensory‐motor and perceptual systems.

This gradient‐based dissociation is consistent with the known topographical organisation of control networks. SCN occupies a position closer to the heteromodal apex of the principal gradient, adjacent to default mode regions, consistent with its role in constraining semantic retrieval in a flexible, context‐sensitive manner (Chiou et al. 2023; Wang et al. 2020). In contrast, MDN lies closer to unimodal sensory and motor systems and showed stronger activation associated with perceptual difficulty, supporting the view that it plays a more prominent role in the control of externally oriented perception and action (cf. Wang et al. 2024). Although MDN itself is largely insensitive to stimulus modality, it is adjacent to regions showing visual and auditory preferences (Assem et al. 2022; Wang et al. 2024), suggesting that the principal gradient may organise a hierarchy of control processes ranging from modality‐sensitive systems proximal to MDN through to highly heteromodal semantic control processes within SCN near the DMN apex.

Consistent with this account, left IFG showed sensitivity to both bottom‐up and top‐down manipulations of semantic control demands. Previous work has demonstrated that left IFG responds to increased demands on controlled retrieval, such as weak versus strong associations, as well as to top‐down instructions to focus on specific semantic features (Badre et al. 2005; Chiou et al. 2018; Davey et al. 2016; Noonan et al. 2013; Whitney et al. 2011). In the current study, left IFG showed greater activation for written words when task goals were known in advance, consistent with the application of top‐down constraints during semantic decision‐making. The top‐down control is likely to influence the processing of Word 1 by allowing more task‐relevant material to be retrieved as well as potentially influencing the decision‐making process at Word 2, since the information retrieved should be better aligned with the link between the words. This is also consistent with an MEG study using the same paradigm, which found that the IFG demonstrated early, top‐down oscillatory responses to prior task knowledge, whereas the pMTG exhibited such effects only at a later, bottom‐up processing stage (Eisenhauer et al. 2024). Behavioral performance (i.e., Accuracy and Reaction time) was observed to be matched for Known and Unknown trials in both modalities, as a result of controlled difficulty across Known and Unknown conditions in our design. This indicates that although proactive semantic retrieval in Known trials elicited stronger left IFG activation, this enhanced activation did not facilitate behavioural performance. Auditory input, by contrast, is inherently more ambiguous; accordingly, an adjacent posterior IFG/insula region showed greater engagement for spoken words when task knowledge was unavailable, potentially reflecting increased demands on control processes that reduce uncertainty during early stages of word recognition. These findings fit with evidence for functional heterogeneity within left IFG, with anterior regions more selectively supporting semantic control and posterior regions contributing to broader aspects of controlled language and perceptual processing (Badre and D'esposito 2009; Demb et al. 1995; Gabrieli et al. 1998). The posterior insula's connectivity with auditory, visual, and sensorimotor cortices further supports its role in coordinating control across multiple processing domains within MDN (Bamiou et al. 2003; Zhang et al. 2019). The pMTG, a region suggested to be a key site in the SCN, was not observed to respond to the Known vs. Unknown contrasts in the present study. This may be due to its specific role in stimulus‐driven semantic control, such as the flexible integration of incoming information within a specific context (Davey et al. 2016; Eisenhauer et al. 2024; Jackson 2021; Lambon Ralph et al. 2017). This interpretation is consistent with our finding that pMTG responded more strongly to Word 2 than to Word 1, reflecting its role in this type of context‐driven control (see Table S1 and Figure S2).

While these distinct aspects of control—that is top‐down semantic control associated with task knowledge, and control demands driven by perceptual difficulty—were separable both psychologically, in terms of their sensitivity to task manipulations, and anatomically, in terms of differential engagement of SCN and MDN, several regions showed more complex response profiles. A cluster in temporal‐occipital cortex bordering visual word form area and MDN supported auditory perception when recognition demands were high (at the first word) as well as semantic decision‐making for written words, when controlled retrieval demands were greater. Similarly, the left posterior IFG/insula showed stronger responses to auditory inputs in the absence of task knowledge and to written inputs when task knowledge was available. One possibility is that these regions lie at the interface between SCN and MDN and therefore contribute to both forms of control, or that distinct effects at the individual level are merged in group analyses. Alternatively, orthographic processes in temporal‐occipital cortex and verbal motor processes in posterior IFG/insula may be recruited during both word recognition and semantic decision‐making (Dehaene and Cohen 2011; Krieger‐Redwood et al. 2015).

Although we utilised the same task paradigm and items for spoken and written words, we examined the effect of modality across two groups of participants; this has the advantage that we can be confident that there is no influence of episodic memory within the modality contrast, but differences in functional organisation between participants might mask fine‐grained functional variation attributable to modality. Moreover, performance was not matched across the auditory and visual tasks: participants indicated that they were not always able to hear the spoken words and they took longer to respond to them. This is consistent with prior work showing increased perceptual and working‐memory demands for spoken input (Booth et al. 2002; Francis and Nusbaum 2009). However, the brain region showing the task knowledge by modality interaction did not show a stronger response to auditory trials in general—instead it showed opposing effects of task knowledge for visual and auditory inputs. For auditory trials, there was a stronger response when the type of knowledge to be retrieved was not cued in advance (i.e., for Unknown vs. Known trials)—and this absence of knowledge might be expected to increase the ambiguity of auditory inputs, making decisions more demanding of control processes. This pattern is consistent with an effect of difficulty in this region, even though the behavioural data did not suggest that Known trials were easier than Unknown. However, for visual trials, there was more activation for Known than for Unknown trials, when the semantic control system could constrain retrieval, and it is not straightforward to explain this pattern as an effect of task difficulty. We therefore interpret the modality effects as reflecting differences in how semantic control is deployed across modalities, potentially due to increased demands on selection and maintenance of task‐relevant semantic features for auditory input, rather than difficulty in a generic sense. While we did not directly manipulate perceptual difficulty for spoken and visual words, the background noise in the MRI scanner would have influenced auditory perception more than visual perception. A direct contrast of the effect of perceptual difficulty across modalities within the same sample would help to confirm whether the stronger activation for auditory inputs in MDN reflected this aspect of difficulty. Furthermore, we used word2vec as a pragmatic and theoretically neutral control for semantic relatedness, rather than as a comprehensive model of conceptual representation. Future work directly comparing distributional and experiential semantic models would be valuable for clarifying how different aspects of conceptual knowledge contribute to the engagement of semantic control networks.

Despite these unresolved issues, our study shows that different aspects of semantic task demands dissociate across sites, networks, and within the principal gradient. We found a dissociation between task demands associated with the top‐down application of task knowledge and with the identification of word meaning from spoken inputs in a noisy environment across SCN and MDN. These results suggest distinct functional roles for cognitive control networks that reflect their location within an axis of functional organisation that extends from unimodal to heteromodal cortex.

## Funding

This work was supported by the European Research Council (Project ID: 771863 – FLEXSEM to EJ).

## Ethics Statement

Ethical approval for this study was granted by the York Neuroimaging Centre at the University of York. Informed consent was obtained from all participants prior to participation.

## Conflicts of Interest

The authors declare no conflicts of interest.

## Supporting information


**Figure S1:** The contrast between visual and auditory tasks (i.e., all four task conditions in auditory modality versus all four task conditions in visual modality). Maps were cluster‐corrected at a threshold of *z* > 3.1 (*p* < 0.05).
**Figure S2:** Mean signal change in each cluster of the semantic control network (taken from Jackson 2021) for Known and Unknown trials relative to implicit baseline, at the first and second words, in visual and auditory tasks.
**Figure S3:** Group‐averaged correlation matrix among all EVs in the auditory modality. EV1:Word1_Category_Known; EV2: Word1_Category_Unknown; EV3: Word1_Thematic_Known; EV4: Word1_Thematic_Unknown; EV5: Word2_Category_Known; EV6: Word2_Category_Unknown; EV7: Word2_Thematic_Known; EV8: Word2_Thematic_Unknown; EV9: Word1_Baseline; EV10: Word2_Baseline; EV11: Word1_Unrelated; EV12: Word2_Unrelated; EV13: Fixations_of_no_interest; EV14: Clues; EV15: Word2vec; EV16: Errors.
**Figure S4:** Group‐averaged correlation matrix among all EVs in the visual modality. EV1:Word1_Category_Known; EV2: Word1_Category_Unknown; EV3: Word1_Thematic_Known; EV4: Word1_Thematic_Unknown; EV5: Word2_Category_Known; EV6: Word2_Category_Unknown; EV7: Word2_Thematic_Known; EV8: Word2_Thematic_Unknown; EV9: Word1_Baseline; EV10: Word2_Baseline; EV11: Word1_Unrelated; EV12: Word2_Unrelated; EV13: Fixations_of_no_interest; EV14: Clues; EV15: Word2vec; EV16: Errors.
**Table S1:** Results of repeated‐measured ANOVAs for mean signal change in each cluster of the semantic control network (taken from Jackson 2021) for Known and Unknown trials relative to implicit baseline, at the first and second words, in visual and auditory tasks.
**Table S2:** Ratings of Co‐occurrence, Physical similarity, Difficulty, and word2vec values for the four Related conditions and the Unrelated trials.
**Table S3:** Group‐averaged correlations among all EVs in the auditory modality.

## Data Availability

The materials and code used to run the study are publicly available on the Open Science Framework (OSF; https://osf.io/swzvr/). Group‐level NIFTI files and network maps are publicly available on NeuroVault in a collection with the title of this article (https://neurovault.org/collections/14750/). The conditions of our ethics approval do not permit public archiving of the raw data supporting this study. Readers seeking access to this data should contact Professor Elizabeth Jefferies, or the local ethics committee at the Department of Psychology and York Neuroimaging Centre, University of York. Access will be granted to named individuals in accordance with ethical procedures governing the reuse of sensitive data. Specifically, the following conditions must be met to obtain access to the data: approval by the Department of Psychology and York Neuroimaging Research Ethics Committees and a suitable legal basis for the release of the data under GDPR.

## Endnotes

^1^
The effect of semantic relation (taxonomic vs. thematic) was not examined. This variable was included only to provide a means of manipulating task knowledge prior to retrieval.
