# Supplementary material for: The Neural Processes Underpinning Flexible Semantic Retrieval in Visual and Auditory Modalities
Source: Hum Brain Mapp. 2026 Apr 22;47(6):e70536. doi: 10.1002/hbm.70536 (PMC13101451; doi:10.1002/hbm.70536)
Supplement: Supplementary file 1 — Figure S1: The contrast between visual and auditory tasks (i.e., all four task conditions in auditory modality versus all four task conditions in visual modality). Maps were cluster‐corrected at a threshold of z > 3.1 (p < 0.05). Figure S2: Mean signal change in each cluster of the semantic control network (taken from Jackson 2021) for Known and Unknown trials relative to implicit baseline, at the first and second words, in visual and auditory tasks. Figure S3: Group‐averaged correlation matrix among all EVs in the auditory modality. EV1:Word1_Category_Known; EV2: Word1_Category_Unknown; EV3: Word1_Thematic_Known; EV4: Word1_Thematic_Unknown; EV5: Word2_Category_Known; EV6: Word2_Category_Unknown; EV7: Word2_Thematic_Known; EV8: Word2_Thematic_Unknown; EV9: Word1_Baseline; EV10: Word2_Baseline; EV11: Word1_Unrelated; EV12: Word2_Unrelated; EV13: Fixations_of_no_interest; EV14: Clues; EV15: Word2vec; EV16: Errors. Figure S4: Group‐averaged correlation matrix among all EVs in the visual modality. EV1:Word1_Category_Known; EV2: Word1_Category_Unknown; EV3: Word1_Thematic_Known; EV4: Word1_Thematic_Unknown; EV5: Word2_Category_Known; EV6: Word2_Category_Unknown; EV7: Word2_Thematic_Known; EV8: Word2_Thematic_Unknown; EV9: Word1_Baseline; EV10: Word2_Baseline; EV11: Word1_Unrelated; EV12: Word2_Unrelated; EV13: Fixations_of_no_interest; EV14: Clues; EV15: Word2vec; EV16: Errors. Table S1: Results of repeated‐measured ANOVAs for mean signal change in each cluster of the semantic control network (taken from Jackson 2021) for Known and Unknown trials relative to implicit baseline, at the first and second words, in visual and auditory tasks. Table S2: Ratings of Co‐occurrence, Physical similarity, Difficulty, and word2vec values for the four Related conditions and the Unrelated trials. Table S3: Group‐averaged correlations among all EVs in the auditory modality. [file HBM-47-e70536-s001.docx]

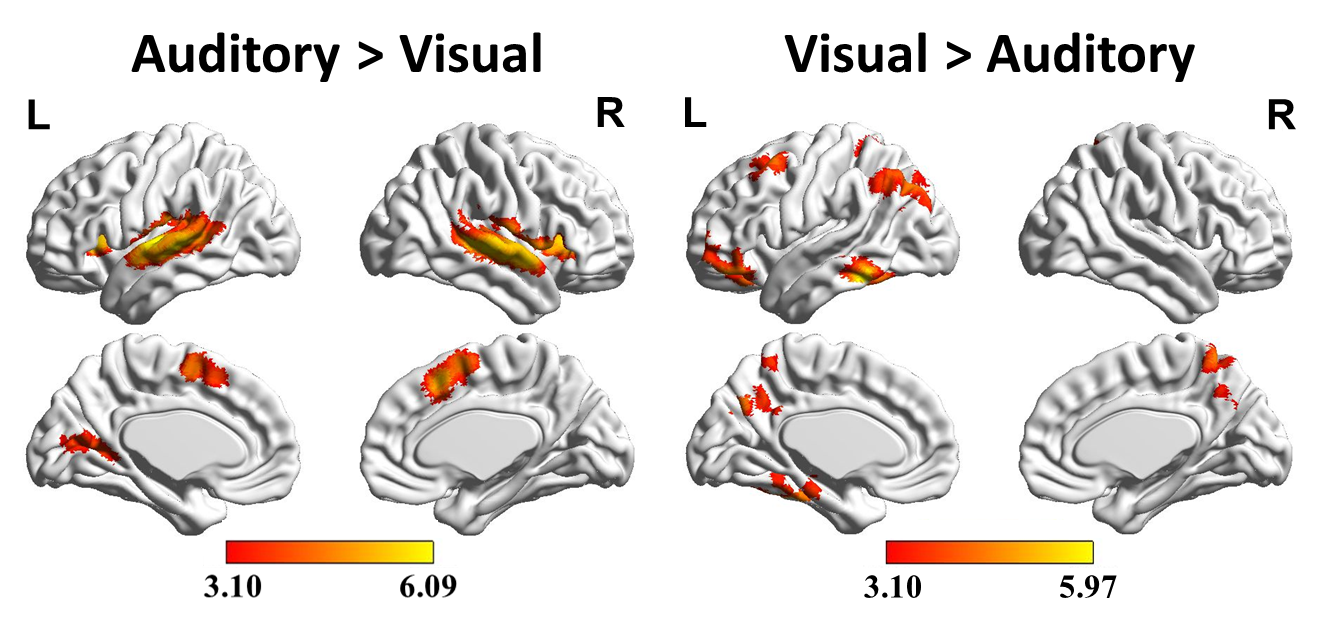


Supplementary Figure S1. The contrast between visual and auditory tasks (i.e., all four task conditions in auditory modality versus all four task conditions in visual modality). Maps were cluster-corrected at a threshold of z > 3.1 (p < .05).


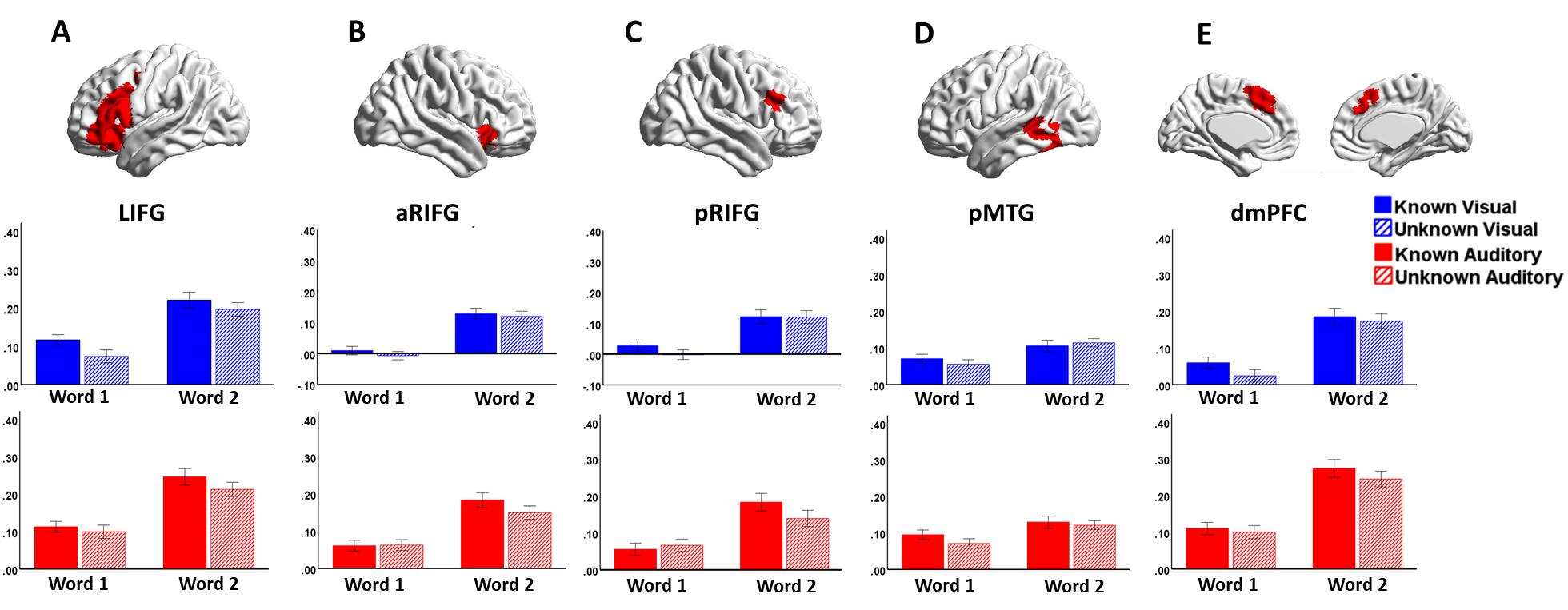


Supplementary Figure S2. Mean signal change in each cluster of the semantic control network (taken from Jackson 2021) for Known and Unknown trials relative to implicit baseline, at the first and second words, in visual and auditory tasks.


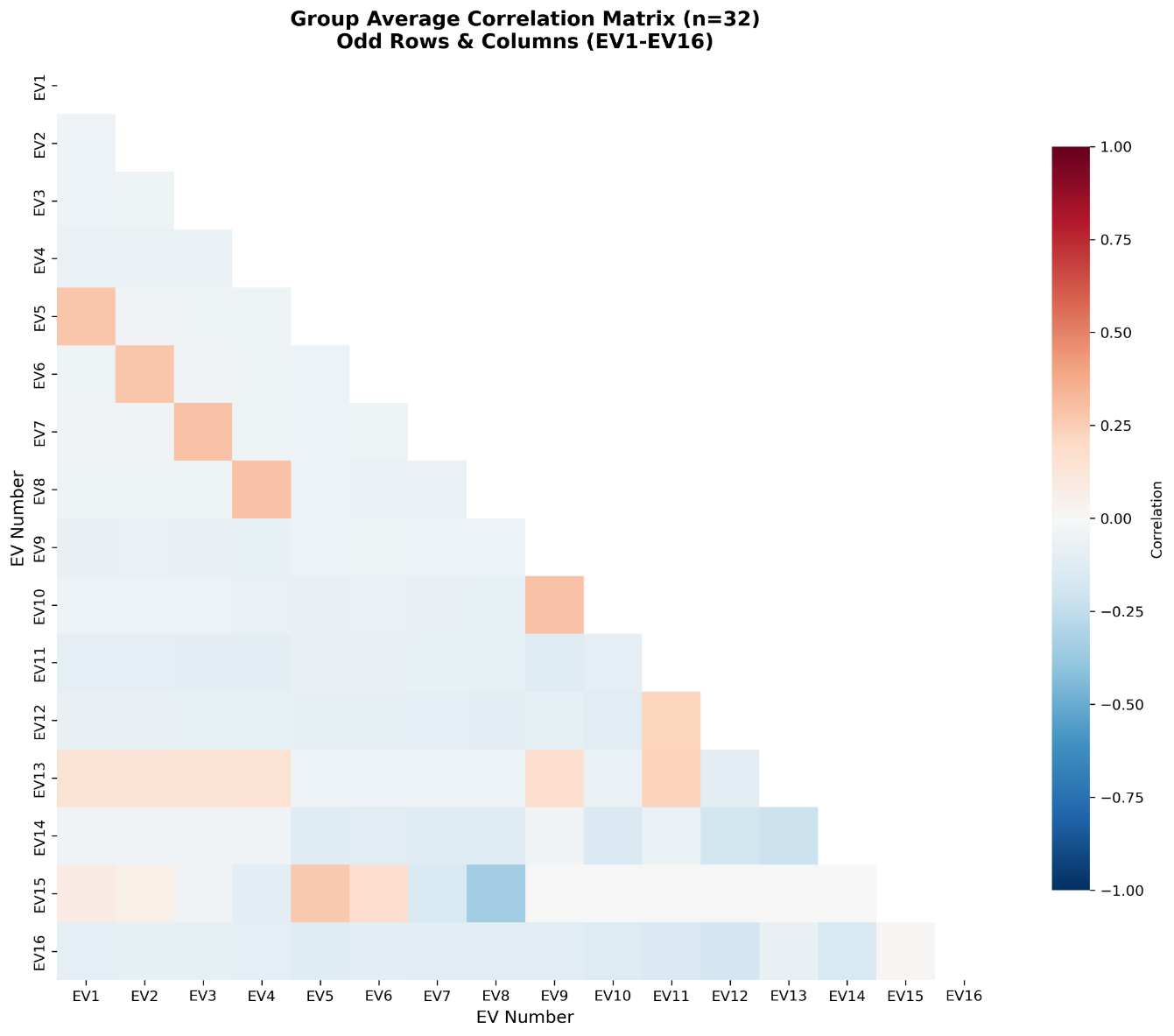


Supplementary Figure S3. Group-averaged correlation matrix among all EVs in the auditory modality. EV1:Word1_Category_Known; EV2: Word1_Category_Unknown; EV3: Word1_Thematic_Known; EV4: Word1_Thematic_Unknown; EV5: Word2_Category_Known; EV6: Word2_Category_Unknown; EV7: Word2_Thematic_Known; EV8: Word2_Thematic_Unknown; EV9: Word1_Baseline; EV10: Word2_Baseline; EV11: Word1_Unrelated; EV12: Word2_Unrelated; EV13: Fixations_of_no_interest; EV14: Clues; EV15: Word2vec; EV16: Errors


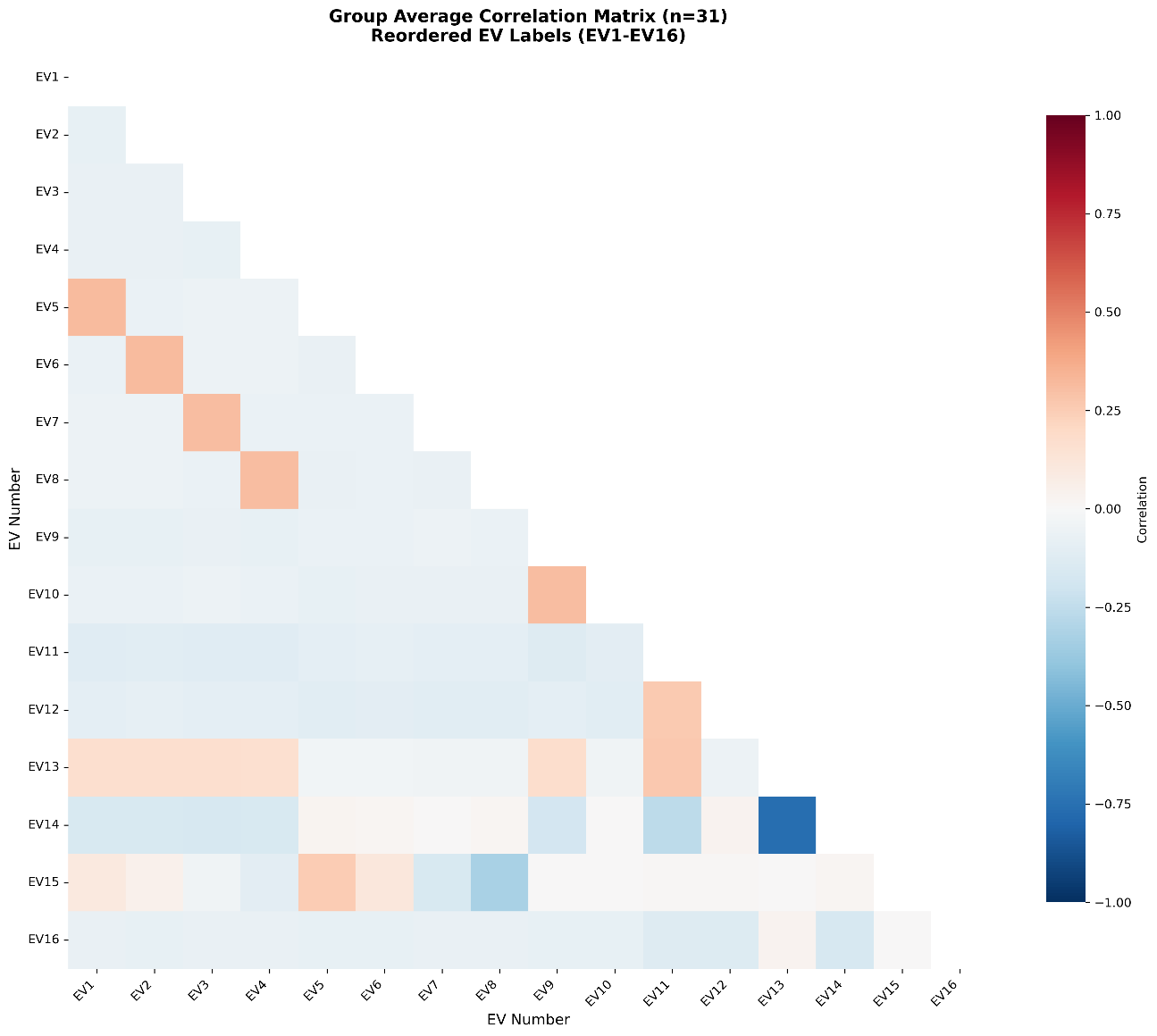


Supplementary Figure S4. Group-averaged correlation matrix among all EVs in the visual modality. EV1:Word1_Category_Known; EV2: Word1_Category_Unknown; EV3: Word1_Thematic_Known; EV4: Word1_Thematic_Unknown; EV5: Word2_Category_Known; EV6: Word2_Category_Unknown; EV7: Word2_Thematic_Known; EV8: Word2_Thematic_Unknown; EV9: Word1_Baseline; EV10: Word2_Baseline; EV11: Word1_Unrelated; EV12: Word2_Unrelated; EV13: Fixations_of_no_interest; EV14: Clues; EV15: Word2vec; EV16: Errors

Supplementary Table S1. Results of repeated-measured ANOVAs for mean signal change in each cluster of the semantic control network (taken from Jackson 2021) for Known and Unknown trials relative to implicit baseline, at the first and second words, in visual and auditory tasks.

|  |  | LIFG | | | aRIFG | | | pRIFG | | | pMTG | | | dmPFC | | |
| --- | --- | --- | --- | --- | --- | --- | --- | --- | --- | --- | --- | --- | --- | --- | --- | --- |
|  |  | F | p | *η^2^* | F | p | *η^2^* | F | p | *η^2^* | F | p | *η^2^* | F | p | *η^2^* |
| Main effect | Modality | F(1,56) = .709 | .403 | .013 | F(1,56) = 10.125 | **.002** | .153 | F(1,56) = 4.749 | **.034** | .078 | F(1,56) =1.413 | .240 | .025 | F(1,56) = 10.666 | **.002** | .160 |
|  | Task knowledge | F(1,56) = 13.710 | **< .001** | .197 | F(1,56) = 4.069 | **.048** | .068 | F(1,56) = 5.600 | **.021** | .091 | F(1,56) = 2.322 | .133 | .040 | F(1,56) = 8.144 | **.006** | .127 |
|  | Word position | F(1,56) = 101.071 | **< .001** | .64 | F(1,56) = 91.26 | **< .001** | .620 | F(1,56) = 58.43 | **< .001** | .511 | F(1,56) = 31.102 | **< .001** | .357 | F(1,56) = 132.541 | **< .001** | .703 |
| Two-way interaction | Modality × Task Knowledge | F(1,56) = .470 | .496 | .008 | F(1,56) = .047 | .828 | .001 | F(1,56) = .005 | .946 | .000 | F(1,56) = 1.020 | .317 | .018 | F(1,56) = .089 | .766 | .002 |
|  | Modality × Word position | F(1,56) = .193 | .662 | .003 | F(1,56) = .650 | .424 | .011 | F(1,56) = .082 | .776 | .001 | F(1,56) = .065 | .800 | .001 | F(1,56) = .471 | .495 | .008 |
|  | Task knowledge × Word position | F(1,56) = .003 | .957 | .000 | F(1,56) = .628 | .432 | .011 | F(1,56) = .553 | .460 | .010 | F(1,56) = 2.340 | .132 | .040 | F(1,56) = .034 | .853 | .001 |
| Three-way interaction | Modality × Task Knowledge × Word position | F(1,56) = 1.245 | .269 | .022 | F(1,56) = 1.638 | .206 | .028 | F(1,56) = 4.892 | **.031** | .080 | F(1,56) = .059 | .809 | .001 | F(1,56) = 1.811 | .184 | .031 |

Supplementary Table S2. Ratings of Co-occurrence, Physical similarity, Difficulty, and word2vec values for the four Related conditions and the Unrelated trials.

| **Conditions** | **Co-occurrence** | **Physical similarity** | **Difficulty** | **Word2vec*** |
| --- | --- | --- | --- | --- |
| Known goal taxonomic relation | 3.11 ± .84 | 4.94 ± .69 | 4.68 ± .79 | .36 ± .09 |
| Unknown goal taxonomic relation | 3.05 ± .76 | 4.8 ± .99 | 4.69 ± .80 | .33 ± .13 |
| Known goal thematic relation | 5.25 ± .88 | 1.67 ± .75 | 5.03 ± 1.31 | .25 ± .10 |
| Unknown goal thematic relation | 5.48 ± .86 | 1.61 ± .83 | 5 ± 1.28 | .21 ± .11 |
| Known goal unrelated condition | 1.23 ± .17 | 1.07 ± .10 | 6.70 ± .18 | — |
| Unknown goal unrelated condition | 1.18 ± .27 | 1.08 ± .12 | 6.77 ± .19 | — |

⁎

The word2vec values were only available for 115 word pairs of the 120 semantic related word pairs, with 1 missing value for Known Goal Taxonomic Relations, Known and Unknown Goal Thematic Relations, and 2 missing values for Unknown Goal Taxonomic Relations.

Supplementary Table S3. Group-averaged correlations among all EVs in the auditory modality.

|  | EV1 | EV2 | EV3 | EV4 | EV5 | EV6 | EV7 | EV8 | EV9 | EV10 | EV11 | EV12 | EV13 | EV14 | EV15 | EV16 |
| --- | --- | --- | --- | --- | --- | --- | --- | --- | --- | --- | --- | --- | --- | --- | --- | --- |
| EV1 | 1 | -0.062 | -0.059 | -0.065 | 0.281 | -0.048 | -0.046 | -0.050 | -0.075 | -0.062 | -0.096 | -0.074 | 0.145 | -0.042 | 0.085 | -0.099 |
| EV2 | -0.062 | 1 | -0.054 | -0.066 | -0.046 | 0.280 | -0.042 | -0.052 | -0.070 | -0.056 | -0.095 | -0.075 | 0.139 | -0.043 | 0.062 | -0.088 |
| EV3 | -0.059 | -0.054 | 1 | -0.067 | -0.047 | -0.043 | 0.291 | -0.053 | -0.076 | -0.061 | -0.103 | -0.079 | 0.135 | -0.041 | -0.042 | -0.092 |
| EV4 | -0.065 | -0.066 | -0.067 | 1 | -0.051 | -0.051 | -0.054 | 0.290 | -0.079 | -0.064 | -0.102 | -0.080 | 0.149 | -0.043 | -0.106 | -0.096 |
| EV5 | 0.281 | -0.046 | -0.047 | -0.051 | 1 | -0.060 | -0.058 | -0.062 | -0.061 | -0.073 | -0.076 | -0.092 | -0.062 | -0.124 | 0.269 | -0.122 |
| EV6 | -0.048 | 0.280 | -0.043 | -0.051 | -0.060 | 1 | -0.053 | -0.064 | -0.054 | -0.070 | -0.075 | -0.093 | -0.055 | -0.117 | 0.164 | -0.106 |
| EV7 | -0.046 | -0.042 | 0.291 | -0.054 | -0.058 | -0.053 | 1 | -0.068 | -0.059 | -0.073 | -0.081 | -0.100 | -0.062 | -0.121 | -0.163 | -0.112 |
| EV8 | -0.050 | -0.052 | -0.053 | 0.290 | -0.062 | -0.064 | -0.068 | 1 | -0.061 | -0.078 | -0.079 | -0.102 | -0.061 | -0.120 | -0.350 | -0.116 |
| EV9 | -0.075 | -0.070 | -0.076 | -0.079 | -0.061 | -0.054 | -0.059 | -0.061 | 1 | 0.292 | -0.117 | -0.092 | 0.167 | -0.043 | 0.004 | -0.114 |
| EV10 | -0.062 | -0.056 | -0.061 | -0.064 | -0.073 | -0.070 | -0.073 | -0.078 | 0.292 | 1 | -0.094 | -0.115 | -0.063 | -0.144 | 0.007 | -0.137 |
| EV11 | -0.096 | -0.095 | -0.103 | -0.102 | -0.076 | -0.075 | -0.081 | -0.079 | -0.117 | -0.094 | 1 | 0.222 | 0.229 | -0.066 | 0.003 | -0.151 |
| EV12 | -0.074 | -0.075 | -0.079 | -0.080 | -0.092 | -0.093 | -0.100 | -0.102 | -0.092 | -0.115 | 0.222 | 1 | -0.106 | -0.192 | 0.005 | -0.182 |
| EV13 | 0.145 | 0.139 | 0.135 | 0.149 | -0.062 | -0.055 | -0.062 | -0.061 | 0.167 | -0.063 | 0.229 | -0.106 | 1 | -0.213 | 0.006 | -0.078 |
| EV14 | -0.042 | -0.043 | -0.041 | -0.043 | -0.124 | -0.117 | -0.121 | -0.120 | -0.043 | -0.144 | -0.066 | -0.192 | -0.213 | 1 | 0.000 | -0.157 |
| EV15 | 0.085 | 0.062 | -0.042 | -0.106 | 0.269 | 0.164 | -0.163 | -0.350 | 0.004 | 0.007 | 0.003 | 0.005 | 0.006 | 0.000 | 1 | 0.009 |
| EV16 | -0.099 | -0.088 | -0.092 | -0.096 | -0.122 | -0.106 | -0.112 | -0.116 | -0.114 | -0.137 | -0.151 | -0.182 | -0.078 | -0.157 | 0.009 | 1 |

EV1:Word1_Category_Known; EV2: Word1_Category_Unknown; EV3: Word1_Thematic_Known; EV4: Word1_Thematic_Unknown; EV5: Word2_Category_Known; EV6: Word2_Category_Unknown; EV7: Word2_Thematic_Known; EV8: Word2_Thematic_Unknown; EV9: Word1_baseline; EV10: Word2_baseline; EV11: Word1_Unrelated; EV12: Word2_Unrelated; EV13: Fixations_of_no_interest; EV14: Clues; EV15: Word2vec; EV16: Errors

Supplementary Table S4. Group-averaged correlations among all EVs in the visual modality.

|  | EV1 | EV2 | EV3 | EV4 | EV5 | EV6 | EV7 | EV8 | EV9 | EV10 | EV11 | EV12 | EV13 | EV14 | EV15 | EV16 |
| --- | --- | --- | --- | --- | --- | --- | --- | --- | --- | --- | --- | --- | --- | --- | --- | --- |
| EV1 | 1 | -0.079 | -0.074 | -0.076 | 0.314 | -0.064 | -0.059 | -0.061 | -0.085 | -0.069 | -0.122 | -0.098 | 0.167 | -0.164 | 0.100 | -0.077 |
| EV2 | -0.079 | 1 | -0.075 | -0.072 | -0.063 | 0.314 | -0.060 | -0.056 | -0.084 | -0.067 | -0.117 | -0.093 | 0.171 | -0.164 | 0.048 | -0.079 |
| EV3 | -0.074 | -0.075 | 1 | -0.081 | -0.059 | -0.060 | 0.312 | -0.064 | -0.077 | -0.061 | -0.121 | -0.096 | 0.166 | -0.166 | -0.043 | -0.076 |
| EV4 | -0.076 | -0.072 | -0.081 | 1 | -0.061 | -0.057 | -0.063 | 0.312 | -0.080 | -0.063 | -0.118 | -0.096 | 0.160 | -0.159 | -0.108 | -0.072 |
| EV5 | 0.314 | -0.063 | -0.059 | -0.061 | 1 | -0.076 | -0.070 | -0.072 | -0.068 | -0.080 | -0.097 | -0.113 | -0.038 | 0.023 | 0.254 | -0.079 |
| EV6 | -0.064 | 0.314 | -0.060 | -0.057 | -0.076 | 1 | -0.070 | -0.066 | -0.066 | -0.077 | -0.093 | -0.108 | -0.037 | 0.019 | 0.116 | -0.081 |
| EV7 | -0.059 | -0.060 | 0.312 | -0.063 | -0.070 | -0.070 | 1 | -0.075 | -0.061 | -0.072 | -0.097 | -0.115 | -0.040 | 0.004 | -0.162 | -0.078 |
| EV8 | -0.061 | -0.056 | -0.064 | 0.312 | -0.072 | -0.066 | -0.075 | 1 | -0.064 | -0.075 | -0.096 | -0.112 | -0.043 | 0.016 | -0.327 | -0.073 |
| EV9 | -0.085 | -0.084 | -0.077 | -0.080 | -0.068 | -0.066 | -0.061 | -0.064 | 1 | 0.309 | -0.126 | -0.101 | 0.177 | -0.181 | 0.001 | -0.083 |
| EV10 | -0.069 | -0.067 | -0.061 | -0.063 | -0.080 | -0.077 | -0.072 | -0.075 | 0.309 | 1 | -0.102 | -0.117 | -0.044 | 0.006 | 0.003 | -0.086 |
| EV11 | -0.122 | -0.117 | -0.121 | -0.118 | -0.097 | -0.093 | -0.097 | -0.096 | -0.126 | -0.102 | 1 | 0.259 | 0.268 | -0.263 | 0.010 | -0.127 |
| EV12 | -0.098 | -0.093 | -0.096 | -0.096 | -0.113 | -0.108 | -0.115 | -0.112 | -0.101 | -0.117 | 0.259 | 1 | -0.062 | 0.036 | 0.015 | -0.134 |
| EV13 | 0.167 | 0.171 | 0.166 | 0.160 | -0.038 | -0.037 | -0.040 | -0.043 | 0.177 | -0.044 | 0.268 | -0.062 | 1 | -0.765 | 0.006 | 0.036 |
| EV14 | -0.164 | -0.164 | -0.166 | -0.159 | 0.023 | 0.019 | 0.004 | 0.016 | -0.181 | 0.006 | -0.263 | 0.036 | -0.765 | 1 | 0.018 | -0.168 |
| EV15 | 0.100 | 0.048 | -0.043 | -0.108 | 0.254 | 0.116 | -0.162 | -0.327 | 0.001 | 0.003 | 0.010 | 0.015 | 0.006 | 0.018 | 1 | 0.006 |
| EV16 | -0.077 | -0.079 | -0.076 | -0.072 | -0.079 | -0.081 | -0.078 | -0.073 | -0.083 | -0.086 | -0.127 | -0.134 | 0.036 | -0.168 | 0.006 | 1 |

EV1:Word1_Category_Known; EV2: Word1_Category_Unknown; EV3: Word1_Thematic_Known; EV4: Word1_Thematic_Unknown; EV5: Word2_Category_Known; EV6: Word2_Category_Unknown; EV7: Word2_Thematic_Known; EV8: Word2_Thematic_Unknown; EV9: Word1_baseline; EV10: Word2_baseline; EV11: Word1_Unrelated; EV12: Word2_Unrelated; EV13: Fixations_of_no_interest; EV14: Clues; EV15: Word2vec; EV16: Errors

**A detailed description of the materials used in the present study has been moved here as the Supplementary Material to improve the readability of the main manuscript.**

## **Materials**

In both the visual and auditory tasks, participants decided whether the probe and target words were semantically related in a semantic relatedness judgement task. Items were linked by one of two different types of semantic relationships only – taxonomic (i.e. they were in the same semantic category) or thematic (i.e. the items were commonly found or used together). On half of the trials, participants were told in advance which relationship would be probed before the presentation of the word pair. For the other half of the trials, participants decided about semantic relatedness based on the two items presented, with no specific instructions in advance. A 2 (Task Knowledge: Known Goal vs. Unknown Goal) × 2 (Semantic Relation: Taxonomic relation vs. Thematic relation) fully-factorial within-subjects design was used to create four conditions, with each experimental condition including 30 related trials. 60 unrelated word pairs were generated without repeating words from the related pairs (i.e., each pair was unique and there was no overlap across conditions). Overall, 120 related and 60 unrelated word pairs were included in this task. We included a greater number of related trials (n=120) to ensure robust modeling and comparison of the BOLD signal across conditions, which is the focus of our hypotheses. The number of unrelated trials (n=60), while smaller, is sufficient to yield a reliable baseline parameter estimate. This asymmetric design allows us to maximize statistical power for the contrasts of interest while keeping the scan session to a prudent length, thereby minimizing participant fatigue and maintaining data quality. The trials were then evenly divided into two sets corresponding to the Known Goal (60 related trials, 30 unrelated) and Unknown Goal (60 related trials, 30 unrelated) conditions.

The assignment of words to conditions was confirmed using an independent sample of 30 participants who provided subjective ratings of thematic relatedness (co-occurrence), taxonomic relatedness (physical similarity), and the difficulty of identifying a connection between the items. Thematically-related word pairs had higher co-occurrence compared to taxonomically-related word pairs, while the taxonomically-related word pairs had higher physical similarity than the thematically-related word pairs. Rated difficulty was the same across both the taxonomic and thematic conditions, and across Known Goal and Unknown Goal trials. For unrelated word pairs, another 12 participants rated Co-occurrence, Physical similarity and Difficulty to confirm the lack of semantic links and equivalence across the Known and Unknown Goal conditions (see Table S2 and Zhang et al., 2021 for detailed statistics). In addition, linguistic properties (i.e., word frequency, length, and imageability) of the probe and target words were matched across conditions (see Zhang et al., 2021 for details). Word2vec was also used to provide a metric of strength of association for each word pair, since Zhang et al. (2021) included this variable as a parametric regressor to investigate the effects of controlled retrieval demands when linking together more weakly related concepts. Word2vec is a measure of semantic distance that is based on the assumption that words with similar meanings occur in similar contexts (Mikolov, Chen, Corrado, & Dean, 2013), and this metric can capture both taxonomic (physical similarity) and thematic (occurrence in similar contexts) relationships (see Zhang et al., 2021 for details).

The auditory task employed the same words as the published visual task to allow their direct comparison (Zhang et al., 2021). All words in the auditory task (including instruction words) were recorded in a female voice using Praat software (www.fon.hum.uva.nl/praat/). Each audio word was supplied as a 16-bit mono WAV file at a sampling rate of 44100 Hz, and the intensity of each word was processed uniformly to 70dB. The volume of the spoken words output from the headphones was set to a safe level.

Both the visual and auditory scanning sessions also included a non-semantic baseline task. The non-semantic baseline task was included to account for low-level sensory processing and motor output, allowing us to isolate the neural correlates specific to semantic cognition beyond these fundamental processes. In the visual session, participants were presented with a pair of meaningless letter strings in succession and were asked to decide if they contained the same number of letters (full details in Zhang et al., 2021). In the auditory session, one spoken number was presented as the probe, followed by two spoken numbers in succession. Participants were asked to decide whether the probe number was equal to the sum of the other two numbers. In both the visual and auditory sessions, there were 30 matching trials and 15 mismatching trials in the baseline task.
